# Engineering Metal–Organic Framework‐Derived Electrochemical Interfaces for Sensitive Chemical and Biosensing Applications

**DOI:** 10.1002/open.70333

**Published:** 2026-11-09

**Authors:** Nithish Kumar M, Prabu S, Munusamy Settu, P. Saravanan, Jothi Vinoth kumar, S. Rajkumar, Krishna Prakash Arunachalam, Sandeep Eswaran Panchu, Hendrik C. Swart

**Affiliations:** ^1^ Department of Electronics and Communication Engineering Advanced Communication Technology Chennai Institute of Technology Chennai Tamil Nadu India; ^2^ Centre for Applied Nanomaterials Chennai Institute of Technology Chennai India; ^3^ Department of Chemistry St. Joseph’s College of Engineering Chennai Tamil Nadu India; ^4^ Centre for Advanced Functional Materials Chennai Institute of Technology Chennai Tamil Nadu India; ^5^ Department of Mechanical Engineering Faculty of Manufacturing Institute of Technology Hawassa University Hawassa Ethiopia; ^6^ Centre for Research and Innovation Academy of Maritime Education and Training (AMET) Deemed to be University Chennai Tamil Nadu India; ^7^ Departamento de Ciencias de la Construcción Facultad de Ciencias de la Construcción Ordenamiento Territorial Universidad Tecnológica Metropolitana Santiago Chile; ^8^ Department of Physics University of the Free State Bloemfontein South Africa

**Keywords:** carbon nanotubes, electrochemical sensors, food safety, graphene

## Abstract

Metal–organic frameworks (MOFs) are emerging as a highly promising scaffold for electrochemical sensing due to their unique structural and chemical properties. Comprising metal ions or clusters linked by organic molecules, MOFs possess a large surface area, adjustable pore sizes, and various functional groups, making them valuable for sensitive and selective analyte detection. Recent studies have focused on leveraging these characteristics to enhance electrochemical sensors applicable in environmental monitoring, medical diagnostics, food safety, and energy systems. MOFs improve electron transfer rates, provide greater active site occupancy, and enhance interactions with target analytes, resulting in increased sensitivity and reduced detection limits. Additionally, MOF‐based composites incorporating conductive materials like carbon nanotubes and graphene address the conductivity challenges of pure MOFs. This review outlines the design, synthesis, and functionalization of MOFs for electrochemical sensing, covering sensing mechanisms, electrode modification strategies, practical applications, and the current challenges and future directions for MOF‐based sensors in next‐generation sensing technologies.

## Graphical abstract

Metal–organic framework composites and derived materials provide tunable electrochemical interfaces for sensitive chemical and biosensing. Their enhanced porosity, active‐site accessibility, electron transfer, and catalytic activity enable selective detection of biological and environmental analytes, while composite engineering and thermal transformation offer improved conductivity, stability, and practical sensing performance.

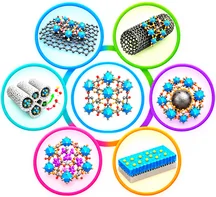

## 1 Introduction

Metal–organic frameworks (MOFs) have been one class of crystalline materials with pores that can be formed by the coordination of metal ions or metal ion clusters with organic ligands. The highly ordered porous structures, high specific surface areas, tunable pore environment, and chemically versatile metal or ligand components have made them useful in a wide range of applications such as gas separation, catalysis, energy storage, drug delivery, and sensing [1, 2]. These features, for sensing, mean that analytes can be enriched at the interface, molecular recognition can occur, and modulation of the chemical or electronic processes at the interface is possible [3]. Because of its high sensitivity, simple instrumentation, swift response, low sample requirements, and compatibility with portable/miniaturized devices, electrochemical sensing has been a subject of great interest [4]. The interleaving of MOFs and electrochemical sensing platforms is particularly fascinating as the porosity of MOFs allows for accumulation and diffusion of the analyte, and the metal centers and functional ligands can participate in the recognition and catalytic processes. The low electrical conductivity and, in some instances, electrochemical and chemical instability of pristine MOFs, however, often constricts the use of these materials in electrochemical sensors. The limitations have inspired the research of conductive MOFs, MOF composites, hybrid MOFs, and MOF‐derived materials [5].

MOF‐based composites and derived materials offer other strategies to address these challenges. The addition of carbon nanomaterials, metals, metal oxides, conductive polymers, and biomolecular materials can enhance the transport of charge, mechanical strength, catalytic activity, and sensing selectivity [6]. Thermal or chemical processing of MOFs can also be used to yield porous carbon materials, metal/metal oxide nanoparticles, and metal–carbon architectures, with a partial preservation of the morphology and porosity of the original framework. It is therefore possible for MOF‐derived materials to inherit properties from the parent material and demonstrate higher conductivity, better electrochemical activity and stability [7]. MOFs and their composites for electrochemical sensing, but most of the literature have been concerned with some specific aspects like general MOF‐based electrochemical sensors, conductive MOFs, MOF composites, MOF‐derived materials, and some specific class of analytes [8]. Therefore, a combination of synthesis, functionalization, thermal transformation, and electrode architecture is important to understand, as this is a more integrated perspective of the structure–property–performance relationship for sensing behavior. More specifically, the relationships between thermal conversion condition and its morphology, porosity and graphitization, chemical state of the metals, accessibility of the active site, diffusion of the analyte, electron transfer, selectivity, and long‐term electrochemical stability need to be systematically considered.

Hence, the current review is limited to the design, synthesis, functionalization, composite engineering and transformation of MOF materials for electrochemical chemical and biosensing applications. The major synthesis methods such as the hydrothermal/solvothermal, microwave‐assisted, and post‐synthetic modification methods are highlighted and their effects on MOF structure and functionality are discussed. MOF–carbon, MOF–metal/metal oxide, MOF–biomolecule, and MOF–polymer hybrid systems are also discussed as well as MOF–based carbonaceous and inorganic materials. The fundamental processes involved in electrochemical sensing such as electron‐transfer pathways, analyte diffusion, active‐site accessibility, adsorption, electrocatalytic reactions, and signal generation are given particular attention [9]. One of the main focus points of this review is the structure–property–performance relationship of MOF‐derived electrochemical sensing interfaces. The influence of the material architecture and thermal conversion conditions on conductivity, porosity, active‐site distribution, selectivity, and electrochemical stability are discussed in relation to the detection of biologically, clinically, and environmentally relevant analytes. The review also examines the possibilities for maintaining or introducing selectivity after structure transformation of the parent MOF, such as by residual porosity, surface chemistry, catalytic sites, and molecular recognition sites. Key analytical parameters, such as linear range, limit of detection, sensitivity, and stability are used for comparison of representative sensing platforms.

Last, the current limitations and challenges in the translation of MOF‐based, and MOF‐derived, electrochemical sensors to practical use are critically discussed. These include batch‐to‐batch reproducibility, scalable synthesis, precursor and processing cost, long‐term stability, matrix interference, real‐sample validation, and integration with flexible, miniaturized, and wearable electronics. In this review, the authors seek to integrate material design and processing with electrochemical mechanisms and device demands, providing a comprehensive view of factors that dictate the design of reliable MOF‐derived electrochemical sensing interfaces and future directions for their improvement.

## 2 Synthesis and Functionalization of MOFs

MOF synthesis is based on the coordination of metal ions or clusters with organic ligands under controlled reaction conditions to produce crystalline architectures with porosity. The chemistry of the nodes and the chemistry of the linkers are the key factors that dictate the basic chemical environment and connectivity of the framework, while factors like composition of the solvent, concentration of the precursors, reaction temperature, reaction time, pH, and concentration of the modulator can affect the nucleation, crystal growth, particle size, particle shape, defect density, or pore structure. Therefore, the synthesis conditions are crucial to influence the structural and physicochemical properties which ultimately dictate the performance of MOFs in electrochemical sensing applications [10]. For electrochemical sensing, the synthesis of MOFs does not necessarily need to result in a crystalline porous framework. This material should also ensure that the analytes have access to the surface, can be chemically functionalized, expose an active site, and be compatible with an electrically conductive electrode surface. Thus, the functional groups can be added by modification of the metal nodes, the organic linkers, the surface of the framework, or all of these. Therefore, functional groups can be added by modification of the metal nodes, the organic linkers, and the surface of the framework or all of these. These modifications can be carried out in the early stages of synthesis, or post‐synthetic methods [11] can be used to form the parent framework.

The nucleation, crystal growth, and morphology of MOFs as well as their functionality can be controlled to a different degree using different‐synthesis strategies. The conventional hydrothermal and solvothermal methods rely on the controlled temperature and pressure to promote the growth of crystals, and they have been commonly used to prepare highly crystalline MOFs. The microwave method of synthesis results in uniform volumetric heating (relatively) rapidly, and it can affect the nucleation and crystal growth and reduce the reaction time. Unlike the post‐synthetic modification, it is a chemical or physical alteration of an already synthesized MOF, which does not require the complete redesign of the MOF’s framework. These strategies can thus complement one another to control the MOF structure and functionality [12, 13].

MOFs are the most attractive in electrochemical sensing since metal nanoparticles or redox‐active centers can be immobilized in their pores. This is not only an increase in electrocatalytic activity but also increases sensitivity and selectivity to trace‐level pollutants or biomolecules [14]. Since the development of MOFs, research has come to an area where the emphasis is no longer on learning their structures but rather on how their morphology and physical properties can affect their performance. Numerous conventional MOFs are produced as microcrystalline powders; however, this may have the drawbacks of low mechanical stability, slow mass transfer, and handling [15]. As a case in point, the MOF powders, when placed in adsorption columns, will compress under pressure, thus becoming resistant to flow.

Initial MOFs were typically constructed out of divalent transition metals (zinc, copper, and cobalt) and formed famous structures, like MOF‐5, HKUST‐1 and ZIF‐8 [16]. In the modern world, the sphere has grown tremendously. Lightweight alkaline earth metals such as magnesium and calcium are now being used to produce MOFs that are more stable and performing. High‐valency metals such as zirconium, titanium, and aluminum are now also used to make MOFs with better stability and performance [17]. Actually, thermodynamic investigations indicate that MOFs constructed with metals of higher valence tend to exhibit stronger metal–ligand connections and enhanced hydrothermal stability. The field of electrochemical sensing has witnessed colossal applications of MOFs [18].

Voltammetry and amperometry are among the techniques frequently employed to detect trace contaminants since they are very sensitive, inexpensive, and respond quickly. Electrochemical platforms are able to sense analytes in parts‐per‐billion limits in the present day, but the efficiency of these devices largely relies on the working electrode. The modification of electrodes is thus essential, and MOFs have some special benefits as electrode modifiers because they are porous, can be chemically tuned and have the capacity to accommodate catalytic species. Appropriate MOF‐based electrodes can significantly increase signal strength, selectivity, and sensing capabilities [19]. Nevertheless, it is difficult to incorporate MOFs into electroanalysis equipment. Most MOFs are not electrically conductive enough, and some are not easily obtainable as uniform films or as stable electrode coatings. Scientists are thus developing MOF composites more and more, incorporating MOFs with conductive nanoparticles such as carbon nanotubes, graphene, or metal nanoparticles [20].

## 3 Hydrothermal Methods

New material development, particularly that of materials with very specific uses, is often reliant on the capability of growing high‐quality crystals [21]. Conventional crystal growth techniques are effective with a wide range of systems; however, they too have shortcomings, especially where low‐temperature conditions are needed [22]. Some materials are not heat‐resistant enough to be subjected to the classical growth methods, and also, there are materials that can experience undesired structural changes or contain defects owing to the thermal stress. At this point, hydrothermal and solvothermal approaches come in as an effective substitute [23]. The special methods enable the cultivation of the crystals at very low temperatures that are well below the melting points of the crystals. Consequently, they lower thermal strain and provide researchers with new phases or single crystals that would otherwise be inaccessible to fairly high‐temperature growth techniques [24]. Figure 1 illustrates the schematic diagram of the solvothermal synthesis of MOFs. Solvothermal and hydrothermal crystal growth are based on the principle of using closed vessels where water or organic solvents have the status of reactive mediums at high temperature and pressure [25]. This low‐temperature climate aids in the slow, gradual development of the crystalline structures. Recognizing the possibility to regulate the reaction environment, the researchers can manipulate the fine balance that is very important during the nucleation and growth mechanism that greatly defines the size, purity, and the overall quality of the resulting crystals [26].

**FIGURE 1:**
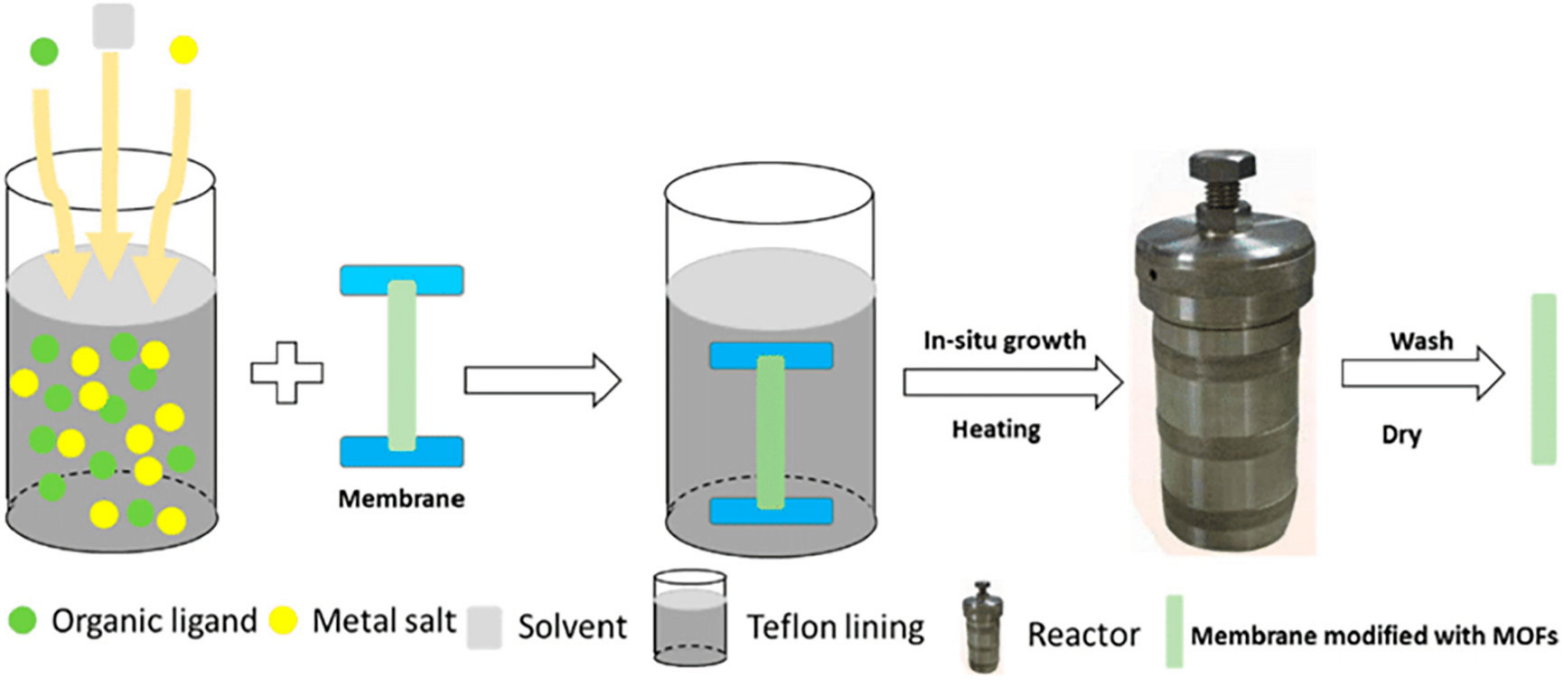
Schematic diagram of solvothermal synthesis of MOFs. Reproduced with permission [20]. Copyright 2018, Elsevier.

The control method proves to be particularly effective with complex materials, such as metal–organic frameworks and coordination polymers. The tremendous scientific enthusiasm behind MOFs and CPs over the past few decades has been explained by their high versatility [27]. MOFs are beautiful in that they are modular, with both their centers of metal and their organic ligand being selectable or tunable to achieve a desired structural or functional behavior. This selectivity enables the development of materials that have selective adsorption properties, catalytic properties, luminescent properties, or magnetic properties [28]. MOFs and CPs are best prepared using hydrothermal and solvothermal processes, particularly those made with carboxylate‐based organic linkers [29]. Carboxylate ligands bind strongly to metal ions and can withstand the high pressure and temperatures of hydrothermal and solvothermal reactions, making them ideal building units for framework construction. Although these methods have been used to synthesize a variety of impressive MOFs and CPs, the final structure is highly dependent on several critical reaction parameters, including solvent, temperature, pH, and reactant ratios [30].

### 3.1 Microwave‐Assisted Approaches

Rapid and efficient heating of the reaction medium results in microwave assisted synthesis as an alternative route to prepare MOFs. The microwave irradiation can directly heat the microwave‐absorbing species in the reaction solution, leading to rapid volumetric heating, which is different from the hydrothermal and solvothermal synthesis for which heat is transferred from the external vessel to the reaction solution [31, 32, 33]. The efficiency of microwave heating is related to solvent’s dielectric properties, composition of the precursors, power of the microwave, temperature, and reaction time [34, 35]. In the microwave‐assisted MOF preparation, the metal precursor and organic ligand are dissolved or dispersed in a suitable solvent, sometimes with modulators or other additives that control the nucleation and crystal growth process. The microwave heating allows for quick heating, which increases coordination between metal ions/cluster and organic ligands, promoting supersaturation and the initiation of initial nuclei [36]. Coordinating and crystal growth around these nuclei finally results in the porous MOF framework. Nucleation and growth processes are highly dependent on the microwave power, irradiation time, concentration of the precursor, composition of the solvent, reaction temperature, and the type of the metal–ligand system used [37, 38].

One of the great advantages of microwave‐assisted synthesis is that the reaction mixture is heated uniformly and very quickly as compared to conventional heating. Microwave irradiation has the potential to affect crystal size and morphology, particle‐size distribution, defect density, and accessible surface area under optimum conditions [32, 36]. These properties can be of special interest for electrochemical sensing as smaller crystallites and accessible pore structures can decrease the diffusion distance for analytes and increase the number of surface and near surface active sites. The physiochemical properties of the product MOF can also be affected by the microwave irradiation conditions, such as the nucleation and crystal growth kinetics. However, higher microwave power or irradiation time does not always lead to better material: higher and uncontrolled heating can lead to crystal growth, aggregation, defect formation, or morphological changes [33, 37]. Thus, the power of the microwaves, the irradiation time, the temperature, the percentage of the solvents, concentration of the precursors, and concentration of the modulator should be optimized together to get the desired structural and functional properties [38, 39].

Compared to conventional synthesis, microwave‐assisted synthesis is especially appealing for the synthesis of MOF nanostructures and materials with controlled morphologies for electrochemical sensing applications. The resultant MOFs can then be combined with other conductive carbon materials, metal nanoparticles, polymers, or other functional parts to enhance their charge transport and electrochemical activity [39]. In this sense, microwave irradiation is not just a faster heating method, but also allows for tuning of the kinetic for the nucleation and crystal growth, which in turn can affect the structural properties that affect analyte diffusion, accessibility of the active sites, and sensing performance. But the disadvantages of the microwave‐assisted synthesis are also reproducibility and scale‐up issues. The absorption of the microwaves is dependent on the solvent, precursor chemistry, reactor configuration, and reaction volume, making it difficult to make direct comparisons between different studies. The variations in microwave power, measurement of the temperature, irradiation time, and reactor geometry may consequently result in variations in MOF morphology and properties. Standardized reporting of microwave power, effective reaction temperature, irradiation time, composition of the solvent, concentration of the precursor, and reactor configuration will thus be helpful in enhancing the reproducibility of the processes and be useful for scaling‐up [34, 35, 40].

### 3.2 Post‐Synthetic Modification and Functionalization

Metal–organic frameworks and their spectacular features, both systematically and comprehensively [41]. What was once a scientific concern with the unusual porous structures of them has grown into a great area of study encompassing numerous disciplines. The usage of MOFs has been explored by scientists over the years, not only in the endeavor to uncover information on their structural properties but also in the effort to harness their great potential in a wide‐scale application. This is because of the flexibility of MOFs in terms of composition, structure, and functionality that allows the researchers to tailor materials with specific properties [42]. The range of approaches to their synthesis is among the primary factors that make MOFs popular [43]. The main goal of initial synthetic processes was the synthesis of crystalline structures whose pore structure is already known [44]. However, as the knowledge of such materials improved, researchers were able to create more advanced synthesis frameworks to maximize stability, porosity, surface area, and chemical activity. Besides these developments, post‐synthetic modification methods have emerged as powerful tools for adding new functionality to MOFs [45].

Such functional and synthetic advancements are reflected in the expansion of the scope of applications of MOFs. Initially, MOFs were investigated in gas adsorption and separation; however, they were also applied in energy‐related applications, including gas storage, fuel processing, and catalysis [46]. With the advancement of the research, MOFs also found their way into the biological and biomedicine fields as they are capable of influencing biological systems in a predictable and controlled way. MOFs have demonstrated specific potential in drug delivery, sensing, imaging, and creation of biologically relevant structures called Bio‐MOFs in life science [47]. They can be used to encapsulate therapeutic agents, protect drugs against early degradation, and release at target sites due to their porous nature. The choice of metal ions and organic ligands enables MOFs to react to biological signals (e.g., pH, enzymes, or redox conditions) to make them more useful in drug delivery systems [48]. MOFs have also shown a lot of potential in biosensing since they possess highly ordered structures and tunable chemical environments [49]. Moreover, imaging agents based on MOFs have also been used to enhance the contrasts of imaging modalities like fluorescence imaging and magnetic resonance imaging with the addition of luminescent ligands or paramagnetic metal cores. BiMOFs emphasize the importance of biodegradable metals and biologically relevant organic linkers to make them as nontoxic as possible and as functional as possible [50].

### 3.3 MOF Composites and Hybrid Nanostructures

Metal–organic frameworks are a special type of porous coordination polymers, obtained by assembling metal‐based nodes and multitopic organic ligands as depicted in Figure 2 [51]. A combination of the inorganic metal centers and organic linkers produces long‐range porous 3D networks that have high surface area and microcrystalline structures [52, 53]. These properties initially led to the attraction of MOFs because the primary application was in gas storage, catalysis, and separation of molecules [54]. These rendered them perfect candidates for the initial applications due to their ability to accommodate as well as selectively connect with the guest molecules inside their pores. With the advancement of research on MOFs, it became more evident that their usage was far beyond these traditional applications [55]. One of the strengths of this type of material is the ease with which the MOF can be rationally designed. The metal ions, organic ligands, or even synthesis conditions can be very specific in the pore size, surface chemistry, and functional properties [56].

**FIGURE 2:**
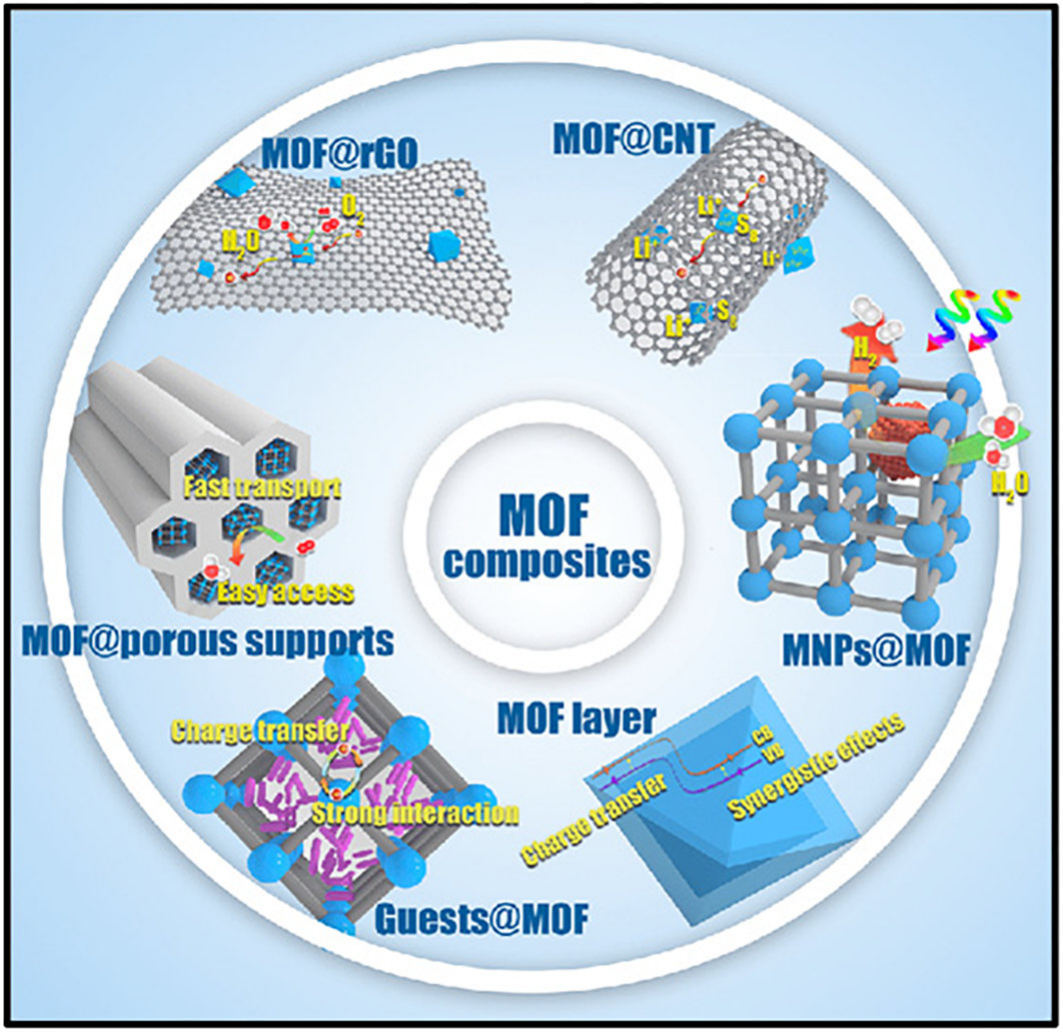
MOFs composites. Reproduced with permission [51]. Copyright 2020, ACS Publication.

Such a vast level of structural and chemical versatility has made MOFs be investigated in a diverse variety of new applications such as chemical sensing and biological systems. To continue optimization of MOF functionality, the focus has been given towards the development of MOF‐based composite materials [57]. In the systems, MOFs are combined with functional elements like organic dyes, biomolecules, nanoparticles, nanowires, nanofibers, or polymers, which add complementary properties. Consequently, MOF substitutes tend to be mechanically stable, efficient in electrical conductivity, and catalytic in comparison to pristine MOFs [58]. The review has presented an extensive description of the current developments in MOF‐based electrochemical as well as optical sensors, based on the discoveries of more than 100 research papers [59].

The specific focus on composite systems is justified by the fact that they have already demonstrated themselves to be particularly useful when it comes to addressing the practical issues of sensor performance, sensitivity, and selectivity. A significant part of the review is devoted to MOF‐based sensors, which include carbon nanomaterials due to their high conductivity in terms of electricity, high surface area, and stability against being easily affected by chemicals [60]. Combined with MOFs, carbon nanotubes, graphene, and graphene‐derived materials are improved in the field of electron transport, charge–transfer dynamics, and accessibility of analytes, leading to strong and highly sensitive sensing platforms. Metal‐ and metal oxide‐MOF composites are also another significant sub‐topic where such nanoparticles as gold, silver, copper, and copper oxide serve as active catalyst sites and enable efficient electron transfer.

### 3.4 MOF–Biomolecule Hybrid Materials

The MOF–biomolecule hybrids possess a combination of the structural and porous properties of MOFs and molecular recognition and biological activities of biomolecules and have proven to be significant class of functional materials for electrochemical biosensing. Enzymes, antibodies, aptamers, peptides and nucleic acids, as well as other bioactive molecules, can be inserted into or attached to MOF structures by adsorption, coordination interaction, covalent attachment, or encapsulation [61]. This hybrid interface can feature the high surface area and tuneable chemical environment of the MOF component with enhanced analyte recognition. The MOF in electrochemical biosensors can act as a porous scaffold to promote biomolecule immobilization and enhance the accessible interfacial area. It can also enhance the local accumulation and diffusion of analytes towards the recognition or catalytic sites, thanks to its porous structure. Simultaneously, the biomolecular part gives specificity for target analyte. Such a combination is especially advantageous when high selectivity is desired in a complex biological matrix in which the intrinsic adsorption or catalytic properties of a MOF alone might not discriminate between structurally similar species [17].

The MOF/biomolecule interaction may also have an influence on the stability and activity of the biological component. Immobilization or encapsulation in an appropriate MOF environment may offer physical containment and contribute to biomolecular dispersion, and the chemical environment of the MOF may have an impact on enzyme activity, substrate access, and electron‐transfer pathways. But if the molecules are too tightly bound or if there are too many interactions between the framework and the biomolecule, the flexibility of the biomolecule or access to the biological active site may also be compromised. Thus, the dimensions of the pores, their functionalization, the loading amount, and the immobilization method should be optimized to ensure good stability of biomolecules and access to the substrate. MOF–biomolecule hybrids can also be combined with conductive materials to circumvent the charge–transfer limitations for electrochemical sensing. In addition to these individual functions, enzyme–conductive MOF or MOF–carbon composites can combine all of them within the same sensing architecture by integrating enzymes, aptamers, antibodies, or nucleic acids and conductive MOFs [38]. Recent advances in MOF–bio molecule systems thus present a paradigm shift from the role of the MOF as a porous framework to the goal of creating integrated bioelectrochemical interfaces, where multiple functions are served by the MOF and bio molecule.

### 3.5 MOF–Polymer Composites

An additional strategy to enhance the practical performance of MOF‐based electrochemical sensing interfaces is the development of MOF–polymer composites. MOFs can be integrated with conductive and functional polymers to enhance the electrical conductivity, mechanical flexibility, adhesion between MOF and polymers, and chemical stability, while retaining the large surface area and tunable porosity of the MOF [40]. Continuous pathways of charge transport can be provided by polymers, such as polyaniline, polypyrrole, PEDOT‐based materials, and other functional polymer matrices, which can be used to integrate MOF materials with electrode surfaces. The polymer part can also represent a catalyst to alter the accessibility and chemical surroundings of MOF surface. The polymer can be either a conductive coating or a bond material that enhances mechanical stability of the sensing film or an interconnected matrix. These architectures can minimize the detachment or aggregation of MOF particles during electrochemical operation and may enhance the durability of modified electrodes. Concurrently, the porous MOF confers extra surface area and adsorption sites, enabling the composite to be both conductive and analytes rich. Polymeric components can offer flexibility and compatibility with the deformable substrates, which makes MOF–polymer composites a highly promising option for flexible and wearable electrochemical sensors. These can then be used in combination with MOFs to create thin, ductile sensing layers for the continuous monitoring of biomarkers and other analytes. Further, the functional groups of polymers can be used to specifically interact with target molecules or to anchor enzymes, aptamers, and other recognition elements [47].

## 4 Electrochemical Sensing Principles

### 4.1 Fundamentals of Electrochemical Sensing

Electrochemical sensing involves the transformation of a chemical interaction or reaction that takes place at the interface between an electrode and an electrolyte, into a measurable electrical signal. In a conventional electrochemical sensing system, the working electrode, the reference electrode, and the counter electrode are inserted in an electrolyte solution containing the analytes to be sensed. Electrochemical reactions at the surface of the working electrode (or in the bulk of the working electrode) can produce a measurable response, which can be correlated with the concentration of the analyte, upon application of an appropriate potential or current. Typically, the sensing process consists of a number of interconnected steps: transport of the analyte from the bulk solution to the electrode–solution interface; adsorption/recognition at the surface of the sensing element; electron‐transfer/electrocatalytic reactions; and generation of an electric signal. Analyte transport can happen by diffusion, migration, and convection; and diffusion can be of significant importance in controlled electrochemical measurements. Thus, the flow rate of the analyte to the electrode and its involvement in the interfacial reaction might affect the sensitivity and response time of the sensor [49].

Electrochemical sensing techniques can be divided into a number of categories with respect to the parameter that is measured. While amperometric and chronoamperometric sensors detect current at a fixed or controlled potential in an electrochemical reaction, voltammetric sensors detect current as a function of potential applied to the electrochemical reaction and can give information about the analyte concentration and the redox processes. As a result, techniques like differential pulse and square wave voltammetry which reduce capacitive background currents have been explored and used to increase sensitivity [59].

The analytical performance of an electrochemical sensor is influenced by the electrode surface to a large extent. Enhancing the surface area available for electrochemically accessible reaction sites and surface functionalization can increase the number of sites at which analyte interaction and electron transfer can occur and can increase molecular recognition and selectivity. But the large physical surface area does not necessarily equate to a large electrochemically active surface area. When designing nanostructured sensing interfaces, it is therefore essential to take into account the pore accessibility, the surface wettability, the conductivity, and the position of the catalytic or recognition sites. Several of these interfacial processes can be modified by MOFs and MOF‐derived materials. The porous structure of MOFs can allow the accumulation and transport of analytes, and chemically active or recognition sites can be provided by the metal nodes or functional ligands [62]. MOF‐derived carbon materials can help to enhance the electrical conductivity, while metal/metal oxide or mixed‐phase derivatives can introduce electrocatalytically active centers. Thus, the sensing response of a MOF‐based electrode is not solely due to the surface area but depends on the mass transport, analyte adsorption, surface recognition, charge transfer, and electrocatalytic activity of the electrode surface. Optimizing one step and disregarding the other(s) may not necessarily enhance sensor performance.

### 4.2 Mechanisms of MOF‐Based Electrochemical Sensing

Metal–organic frameworks represent a rapidly developing group of porous hybrid materials that have been of great research interest in the last several decades [63]. MOFs consist of inorganic metal nodes or clusters with organic ligands to create longer crystalline porous networks [64]. Due to the functional effects of the groups of metal nodes and organic linkers, MOFs can be specifically designed for a given task, which increases their selectivity and specificity in various fields, including electrochemical sensing [65, 66]. Their large surface area and the ability to modify pore environments have been widely used in a vast array of applications in MOFs, such as gas adsorption, catalysis, drug delivery, molecular separation, and sensing. Their new redox‐active electrode materials status has only increased their applicability in electrochemical technologies, especially when used in conjunction with metal nanoparticles that amplify electrocatalytic activity and signal transduction [67, 68, 69].

In spite of the fact that the first research work was conducted mainly to learn about the MOF structures and physicochemical characteristics, the latest investigations are more focused on the connection between morphology, structure, and practical performance [70]. Nonetheless, the majority of MOFs are prepared as microcrystalline powders that have disadvantages, including low mechanical stability, slow mass transfer, and handling and recovery [71]. The metal ions used are an important determinant of the stability and performance of MOF. Although some of the earliest MOFs used divalent metal ions, including zinc, copper, and cobalt, more highly valent metal‐based MOFs (e.g., aluminum, iron, zirconium, and titanium) have been demonstrated to have better hydrothermal and structural stability [72, 73, 74, 75]. MOFs based on aluminum are of special interest as they are low‐cost, abundant, lightweight, and can be produced in large‐scale [76]. Electrochemical sensing techniques are widely favored because of their low cost, high sensitivity, and ability to detect trace analytes using simple instrumentation [77]. MOFs have emerged as promising electrode modifiers due to their high surface area, tunable porosity, and abundance of active sites, enabling sensitive and selective detection of pollutants, heavy metals, and biomolecules [78, 79, 80].

### 4.3 Electron Transfer Pathways

Their high‐order and adaptable structures allow easy diffusion of guest molecules on the basis of interconnecting pores, thus being highly desirable in molecular recognition and transport studies [81, 82, 83, 84]. Due to these properties, MOFs have found extensive applications in traditional applications like the separation of gases, heterogeneous catalysis, and molecular separation [85]. In addition to the conventional applications, multifunctionality based on optical, redox‐active, and magnetic metal centers has shown that it is possible to apply MOFs in complex technologies such as chemical sensing and bioimaging [86]. These features render MOFs versatile systems that can integrate recognition and signal transduction with structural selectivity in a single system [87].

The use of electrochemical analysis methods is common because of their simplicity, reliability and sensitivity and is used in the environment, biomedical diagnostics, and industrial analyses [88]. Although the prospects of MOFs in electrochemical systems were high, the initial literature review identified that only a handful of MOF‐related experiments were devoted to electrochemical or electroanalytical applications [89]. MOFs have a porous structure that is vital in electrochemical functionality due to their ability to selectively transport desired analytes of different sizes and shapes and also increases the host‐guest interactions [90]. Nonetheless, most of the traditional MOFs have a limitation of poor intrinsic electrical conductivity because of poor orbital overlap among metal centers and organic ligands, which restricts long‐range charge transport [91]. To counteract these drawbacks, methods such as the addition of conductive guest species, synthesis of MOF composites and deposition of MOFs onto conductive substrates have been devised to improve electrochemical performance [92].

## 5 Active Site Accessibility

Accessibility of the active sites of MOFs has a significant impact on their electrochemical characteristics in the sensing interfaces. While MOFs have large number of structurally tunable sites and high specific surface area, they do not always have the same number of accessible sites as there are structurally tunable ones, and not every accessible site is necessarily involved in electrochemical reactions [93]. The active sites can be on the external surface of the framework, on the pore walls, on the pore openings, on defects, on organic linkers, or on metal nodes. So, the sensing performance is not only a function of the density of active sites but also of the spatial distribution, the chemical accessibility, and the interaction with the interface of the electrode. The degree of utilization of an active site is one of the most important factors to consider in the diffusion of analytes. It is important that the molecules of the analyte be brought to the sensing layer from the bulk electrolyte and finally to pores or channels that are accessible to the analytes, before the analyte molecules can interact with the internal active sites. This process is affected by the size and connectivity of pores, sensing layer thickness, surface chemistry, size and charge of the analyte, and the composition of the electrolyte [94]. Mass transport can be provided by interconnected micro and mesoporous structure, which can also increase the probability of the analyte molecules reaching internal active sites. Conversely, too thick a sensing film or particle aggregation and blocked pores can increase the diffusion resistance and decrease the number of active sites being effectively used [95].

Accumulation and selectivity of analytes can be additionally modified by the chemical environment inside MOF pores. Analytes can be attracted to pore walls by hydrogen bonding, electrostatic interactions, coordination, and other noncovalent interactions. These interactions can cause enhanced adsorption of the target analyte at the electrochemically active surface [96]. If the adsorption is too strong, however, the desorption of the analytes can be difficult, and the number of active sites may be restricted. Hence, a compromise between analyte enrichment, pore accessibility, and fast mass transport is necessary for the development of an effective MOF‐based sensing interface.

The catalytic interface is the location where the analyte, active sites, electrolyte, and conductive electrode are coming into contact with one another during the sensing process. The electrode is the medium through which electron transfer takes place while analyte adsorption and electrocatalytic reactions can take place at metal centers, redox‐active ligands, defects, and functional groups [97]. Therefore, the catalytic activity of the MOF‐based electrode will rely on whether the catalytically active sites are exposed to the analyte and also electronically connected to the electrode. The electrical conductivity is thus an important consideration in determining the effective utilization of the MOF active sites. Poor intrinsic conductivity in many pristine MOFs is due to poor electronic coupling between the metal centers and the organic linkers. The incorporation of conductive carbon materials, metallic nanoparticles, conductive polymers, or conductive electrode substrates may offer further electron‐transport pathways and facilitate communication of the active sites and the electrode [98]. Hybrid architectures are therefore possible to combine MOF’s high surface area and high chemical tunability with better charge–transfer characteristics. The architecture and thickness of the sensing layer also affect the ratio of active‐site density and analyte transport. The more MOF is applied to an electrode, the more adsorption sites and catalytic sites will be available; however, a greater amount may result in either a thicker layer or a higher density of MOFs deposited on the electrode, both of which will increase the charge–transfer resistance and the diffusion path length. Likewise, MOF particles may aggregate and obstruct the opening of pores and reduce the accessible fraction of the inner surface area for analytes. Therefore, it is not always possible to achieve maximum electrochemical performance by maximizing the amount of MOF material.

These are important factors especially when MOFs are incorporated into flexible and miniaturized electrochemical devices. The sensing layer should have adequate active‐site accessibility and transport of the analyte and have intimate contact with the conductive substrate and be mechanically stable during operation in these systems [99]. In view of this, rational design of MOF‐based sensing interfaces should be based on a multifaceted approach that accounts for pore architecture, exposure of active sites, diffusion of analytes, electrical connectivity, and adhesion of electrode materials.

## 6 MOF‐Derived Nanomaterials as Electrochemical Platforms

Metal–organic frameworks (MOFs) represent an interesting category of hybrid materials that are made of metal centers, inorganic, and organic ligands [100, 101]. This unusual combination gives them the ability to use the structural strength of an inorganic molecule, coupled with the chemical versatility of an organic molecule [102]. Figure 3 demonstrates MOFs that serve as precursors for producing nanocarbon and related nanomaterials. MOFs themselves, having initially been given attention as a promising material in the 1990s, have grown fast to become one of the most sought‐after material families [103]. Their significance is indicated by the fact that, to date, over 90,000 scientific papers regarding MOFs have been published, and the list is steadily expanding at a rate that is quite impressive [104].

**FIGURE 3:**
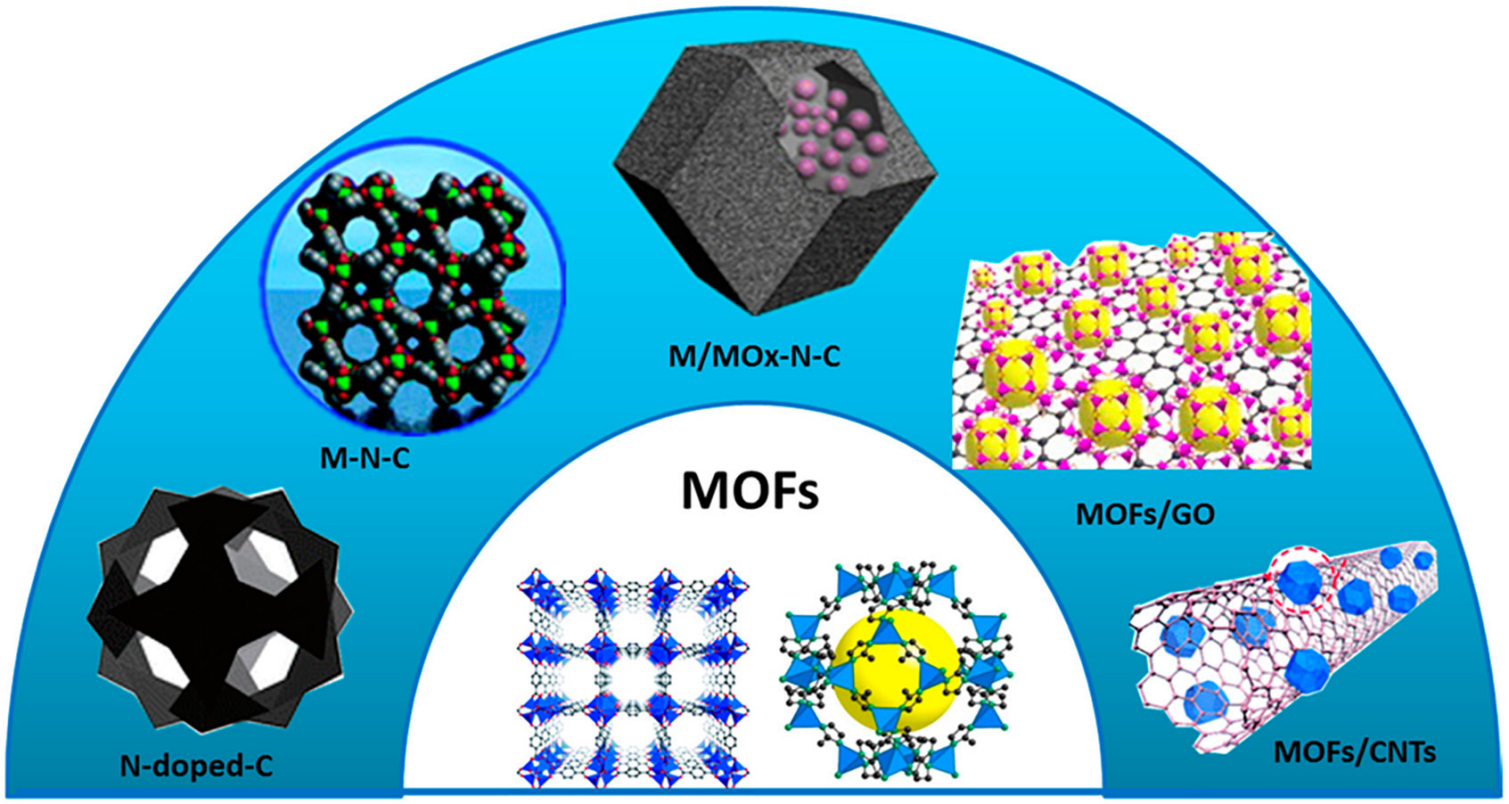
MOFs serve as templates for producing functional nanomaterials. Reproduced with permission [100]. Copyright 2016, MDPI.

The highly tunable nature of MOFs is one of the major factors contributing to the level of interest evoked by these systems [105]. By using the correct selection of metal nodes and organic linkers, scientists could prepare MOFs with a particular pore size, surface area, and chemical properties. This structural versatility has enabled them to be used in gas storage and separation, catalysis, drug delivery, sensing, and environmental remediation. Despite such exotic advantages, pristine MOFs also exhibit certain demerits, in particular, their low conductivity and low stability, especially in the context of electrochemical use [8]. The focus of the modern society is electrochemical technologies, which are employed in the energy conversion and storage systems and electrochemical sensors in health care, environmental and industrial processes monitoring [106]. Even though MOFs have large surface areas and chemical densities, they do not achieve the demanding electrochemical performances of conductivity and long‐term stability, which has prompted efforts to improve performance protocols extensively [107].

Towards therapeutic advantage, much focus has been placed on MOF‐derived materials, where MOFs are used as sacrificial templates in the thermal or chemical transformation processes [108]. This approach allows one to retain the positive structural attributes like porosity and morphology, besides endowing superior electrochemical characteristics. Many MOF‐derived materials, such as metals, metal oxides, metal sulfides, phosphides, and carbon‐based substances, have been shown to conduct better and have better catalytic activity than pristine MOFs [109]. This has seen MOF‐derived materials become highly promising candidates towards electrochemical sensing applications because they have shown enhanced sensitivity, stability, and selectivity to a given analyte [110].

## 7 Effect of Thermal Conversion Conditions

Thermal conversion of MOFs is an effective method to convert the crystalline porous framework to conductive carbonaceous materials, metal/metal oxide nanostructures and metal–carbon composites. In this process, the organic ligands break down or carbonize, and the metal nodes can be transformed into other inorganic forms (such as metal or metal oxide) or remain as metals depending on the temperature [61]. Hence, the morphology, porosity, carbon structure, metal chemical state, and, consequently, the electrochemical sensing performance of the resultant material will be influenced by the temperature, heating rate, treatment time, and atmosphere.

The effect of the calcination temperature on the material is as follows. The calcination temperature has a significant effect on the breaking down of organic linkers, degree of carbonization, crystallinity of inorganic phase, pore formation, and particle growth. Partial decomposition of the organic ligands could take place at relatively low temperatures, leaving behind organic residues and less graphitized. Temperature elevation is conducive to ligand decomposition and carbonization and can enhance the electrical conductivity of carbon derivatives. Higher temperatures can also encourage the crystallization or formation of metal/metal oxide phases. Too much thermal treatment can lead to a reduction in framework size, pore collapse, aggregation of particles, and reduction in functional groups on the surface. Thus, there is usually an optimum temperature needed to meet requirements for structural preservation, accessible porosity, conductivity, and exposure of the active sites. The effect of heating rate and treatment time is as follows. The presence of gas molecules in the MOF structure is accompanied by the release of gases during decomposition of organic ligands, and the rate of this decomposition is controlled by the heating rate. Slow heating rate can allow slow decomposition and reduce sudden breakdown in the structure; this can help to maintain the parent morphology and promote interconnected pore formation. However, if the heating rate is too high, gas will evolve quickly and damage will occur in the local structure, which may cause irregular pore formation or aggregation. The amount of carbonization/crystallization also depends on the length of thermal treatment [67]. Therefore, the heating rate and the holding time must be optimized simultaneously with the temperature, so the two are not independent optimization parameters for each other.

### 7.1 Selectivity and Matrix Interference in MOF‐Derived Electrochemical Sensors

Thermal conversion of MOFs is an effective method to convert the crystalline porous framework to conductive carbonaceous materials, metal/metal oxide nanostructures, and metal–carbon composites. In this process, the organic ligands break down or carbonize, and the metal nodes can be transformed into other inorganic forms (such as metal or metal oxide) or remain as metals depending on the temperature. Hence, the morphology, porosity, carbon structure, metal chemical state, and, consequently, the electrochemical sensing performance of the resultant material will be influenced by the temperature, heating rate, treatment time, and atmosphere. The effect of the calcination temperature on the material is as follows [69]. The calcination temperature has a significant effect on the breaking down of organic linkers, degree of carbonization, crystallinity of inorganic phase, pore formation, and particle growth. Partial decomposition of the organic ligands could take place at relatively low temperatures, leaving behind organic residues and less graphitized. Temperature elevation is conducive to ligand decomposition and carbonization and can enhance the electrical conductivity of carbon derivatives. Higher temperatures can also encourage the crystallization or formation of metal/metal oxide phases. Too much thermal treatment can lead to a reduction in framework size, pore collapse, aggregation of particles, and reduction in functional groups on the surface. Thus, there is usually an optimum temperature needed to meet requirements for structural preservation, accessible porosity, conductivity, and exposure of the active sites [76].

### 7.2 Stability of MOF‐Derived Sensing Interfaces

The stability of an electrochemical sensing interface over extended periods of time is critical, especially if measurements are taken under variable pH, high ionic strength, or extended periods of electrochemical activity. While the conversion of MOFs into carbonaceous materials, metal/metal oxide phases, and metal–carbon composites may enhance the electrochemical stability in comparison to pristine MOFs, they are not necessarily free from structural and chemical degradation. They are highly dependent on the composition of the derived phase, degree of carbonization, the dispersion of the metal, the adhesion of the electrodes, the composition of the electrolyte, and the working potential. In an acidic or alkaline environment, the metal‐containing components can dissolve, change oxidation state, or undergo surface reconstruction [81]. These processes can change the number and chemical characteristics of electrochemically active sites and hence their sensing response on repeated measurements. The stability of the supported metal nanoparticles or metal oxides in the carbon matrices may thus be different than that of the unsupported phases. A carbonaceous framework can offer physical containment and enhance resistance to aggregation or dissolution of the particles but can, over time, be influenced by aggressive electrolytes, modifying the surface chemistry or causing loss of catalytically active species.

Another important parameter is the electrolyte composition which plays a role in the operational stability. The electrical double layer, ion transport, and interfacial adsorption processes can be altered by high concentrations of salts. Competitive adsorption, by ions in the electrolyte, can change the availability of active sites, and extended residence times under high ionic strength may lead to changes in surface charge and interface structure [111]. In biological and environmental samples, other organic species and macromolecules can also adsorb at the surface of the electrode leading to surface fouling and eventual reduction of the available electrochemical surface area.

## 8 Applications of Electrochemical Sensors

The electrode surfaces were extensively altered using a variety of materials in order to provide high sensitivity and some chemical specialization that were not possible at the original electrode. Using MOF bulk crystals with electrocatalytic activity for modification on the electrode surfaces, some groups have recently tried to detect tiny amounts of chemicals due to their high surface areas, particular adsorption affinity, different pore diameters, and bare active sites. To overcome the drawbacks, such as low electronic conductivity, poor stability in water, and inadequate electrocatalytic ability, MOFs have been developed that incorporate additional highly conductive and mechanically robust components in addition to directly altering single component MOFs on the electrode surfaces [87]. Comparison of MOF‐based electrochemical sensors is tabulated in Table 1.

**TABLE 1:** Comparative electrochemical performance of representative MOF/MOF‐derived sensors.

Analyte	MOF‐derived material	Electrochemical method	Linear range	LOD	Sensitivity	Stability	Applications	References
Dopamine	2D Cu‐based conductive MOF	Electrochemical sensing	5.0 × 10^−8^–2.0 × 10^−4^ M	5.1 nM	NR	NR	Chemical sensing	[112]
Dopamine	HKUST‐1/ERGO	DPV	0.2–300 μM	0.013 μM	NR	NR	Chemical sensing	[113]
Dopamine	ZIF‐90/Pt‐Rh	Electrochemical sensing	NR	1 nM	NR	NR	Chemical sensing	[114]
Dopamine	Ag/ZIF‐67	Electrochemical sensing	NR	0.05 μM	NR	NR	Chemical sensing	[114, 115, 116]
Glucose	CoMnP@Co_3_O_4_/CuO	Electrochemical sensing	NR	NR	NR	NR	Glucose sensing	[117, 118, 119]
Glucose	CuCo sulfide derived from MOF	Electrochemical sensing	NR	NR	NR	NR	Glucose sensing	[118, 119]
Glucose	Ni_2_P/graphene	Electrochemical sensing	NR	NR	NR	NR	Nonenzymatic glucose sensing	[120]
Uric acid	CuNi‐MOF@rGO	Electrochemical sensing	10–1000 μM	9.09 μM	0.026 μA μM^−1 ^cm^−2^	NR	Wearable sweat sensing	[121]
As^3+^	Fe‐MOF@mFe_3_O_4_@mC	DPV	0.01–10 nM	6.73 pM	NR	Repeatability: ~4.66%	Water/environmental monitoring	[122, 123]
Lactate	Fe/Co‐MOF/PVA–chitosan hydrogel	Electrochemical oxidation	NR	NR	NR	NR	Simulated sweat	[124]

### 8.1 Detection of Dopamine

Metal–organic frameworks are an outstanding possibility for the detection of dopamine at ultralow concentrations by electrochemical methods. The alteration of electrodes with diverse types of MOFs results in a reduction of the electrode potential of the analytes and boosts redox kinetics, hence increasing the electrochemical sensing of DA. Copper‐based metal–organic frameworks have been extensively employed to enhance the glassy carbon electrode (GCE) for the sensitive and selective electrochemical detection of dopamine (DA) with increased electrochemical activity [125]. A 2D conductive metal–organic framework was engineered utilizing the redox‐active organic linker 2,3,6,7,10,11‐hexahydroxytriphenylene in conjunction with copper metal centers to construct an electrochemical sensor for the detection of dopamine within a concentration range of 5.0 × 10^−8^–2.0 × 10^−4 ^ M, achieving a detection limit of 5.1 nM. The minimal detection limit of DA was mostly due to the π‐conjugated MOF exhibiting strong electrical conductivity and superior charge storage capacity, along with the redox‐active linker possessing significant conjugation, facilitating charge transfer between the metal nodes and organic linkers [112].

Furthermore, the integration of MWCNTs into functionalized MOFs can enhance sensitivity and reduce the detection limit by an order of magnitude, owing to improved electron‐transfer kinetics. A nanocomposite of electro‐reduced graphene oxide (ERGO) and copper‐based MOF HKUST‐1 was recently synthesized via electrochemical deposition, demonstrating superior electrochemical activity towards dopamine (DA) attributed to its increased surface area, high electrical conductivity of ERGO, and optimal porosity. The engineered electrochemical sensor demonstrated a minimal detection limit of 0.013 μM and an extensive linear sensing range from 0.2 to 300 μM. A copper terephthalate MOF‐GO nanocomposite was synthesized via π−π stacking, Cu−O coordination, and hydrogen bonding, demonstrating the synergistic effect of electron mediation from copper terephthalate and reduced graphene [113].

Wang et al. synthesized a hybrid CuCo_2_O_4_@carbon nanocomposite on 3D porous carbon utilizing a Cu‐Co‐ZIF precursor, resulting in the development of a molecular imprinted electrochemical sensor that demonstrated a sensitivity of 720.8 mA mM^−1 ^cm^−2^ and a detection limit of 0.16 mM. The DPV curves indicated an enhancement in the current responsiveness of the MIPs/CuCo_2_O_4_@carbon/3D‐KSC integrated electrode with increasing DA concentration (Figure 4a). The chitosan film was electrodeposited onto CuCo_2_O_4_@carbon/3D‐KSC, resulting in the formation of MIPs [126].

**FIGURE 4:**
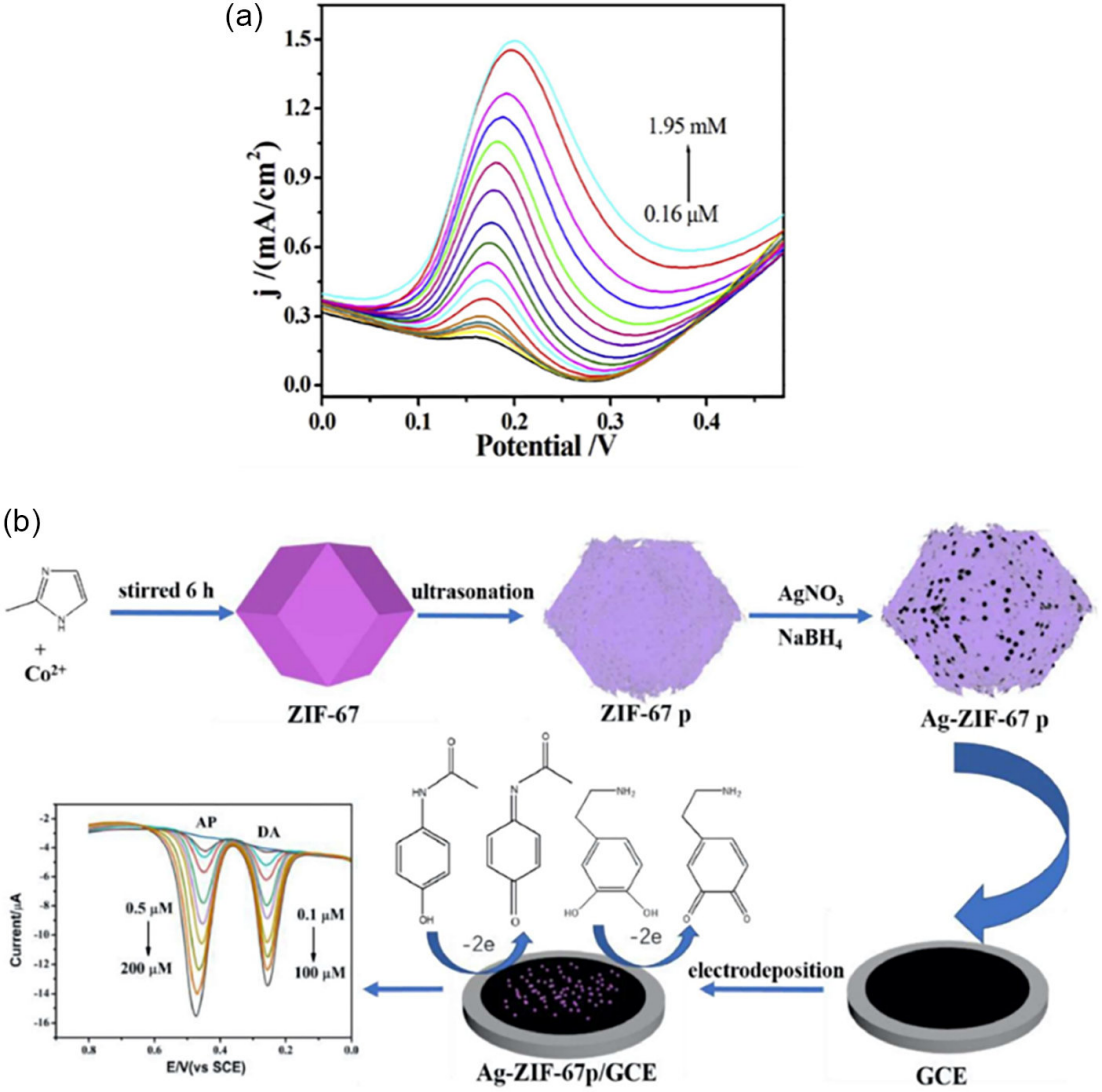
(a) Differential pulse voltammetry. Reproduced with permission [126]. Copyright 2020, Elsevier. (b) Development of Ag‐ZIF‐67 nanopinna‐modified glassy carbon electrode and its utilization in the detection of acetaminophen and dopamine. Reproduced with permission [115]. Copyright 2020, Elsevier.

Noble metal MOF composites augment dopamine detection by enhancing conductivity, catalytic efficacy, and electron transfer. A nanocomposite consisting of ZIF‐90 MOF and the Pt_41_Rh_59_ alloy nanocatalyst was developed for the electrochemical detection of dopamine, achieving a low detection limit of 1 nM. The synthesized MOF contained a free aldehyde group in the organic ligand, enabling covalent binding and adsorption of dopamine. Similarly, Tang et al. generated a silver NP‐decorated ZIF‐67 composite through the in situ reduction of AgNO_3_ precursor on the surface of the MOF, as illustrated in Figure 4b. The synthesized nanocomposite exhibited numerous active sites, elevated conductivity, and superior catalytic characteristics, hence improving the electrochemical performance of the dopamine sensor, which achieved a detection limit of 0.05 μM [114, 115, 116].

### 8.2 Detection of Glucose

Materials generated from MOFs, including metal oxides, metal sulfides, metal phosphides, and layered double hydroxides (LDHs), exhibit significant potential in the advancement of electrochemical glucose sensors. The materials formed from MOFs are highly favored for their capacity to maintain structural stability and porosity following their transformation from MOFs to MOF‐derived materials [117]. A sensor utilizing a CoMnP@Co_3_O_4_/CuO hybrid nanostructure was produced with metal–organic frameworks as a template. The template‐driven material generated from MOFs exhibits increased sensitivity to glucose, likely attributable to the availability of additional active sites, rapid ion transport, and improved catalytic properties. A separate study developed a CuCo sulfide structure for the electrochemical detection of glucose using MOF precursors, while preserving the porous architecture and elevated surface area. Figure 5a illustrates the shape and sensing capabilities of the synthesized CuCo sulfide, indicating its effective response to glucose detection [118]. The preserved porous structure enhanced the material’s conductivity and facilitated the transport of electrolytes and analytes, hence augmenting the electrocatalytic activity towards glucose [119].

**FIGURE 5:**
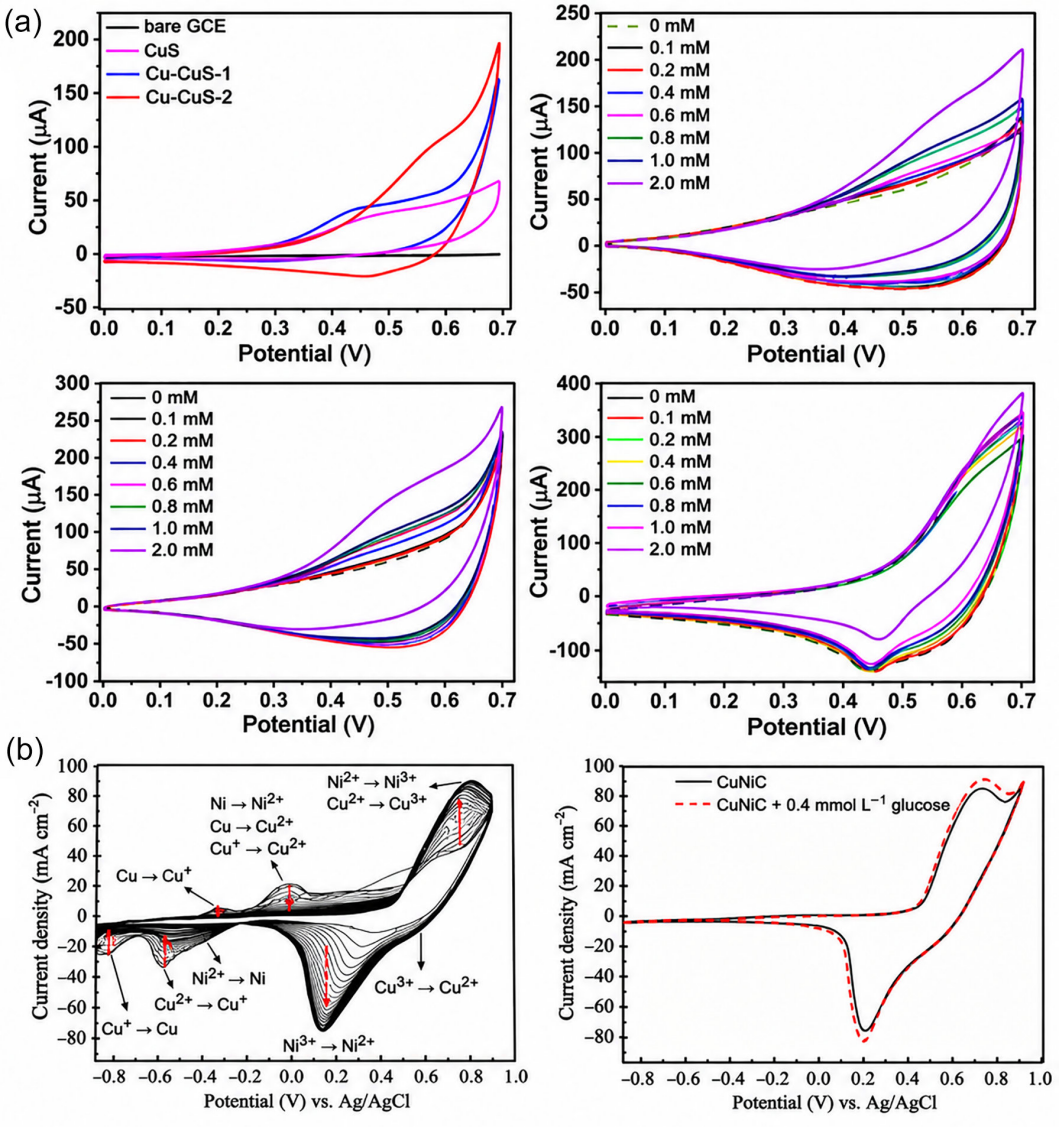
(a) Electrochemical efficacy of copper cobalt sulfides in relation to glucose. Reproduced with permission [118]. Copyright 2022, MDPI. (b) Glucose sensing of CuNi/C in 0.1 M NaOH. Reproduced with permission [120]. Copyright 2018, Springer.

A Ni_2_P and graphene film composite was synthesized utilizing metal–organic frameworks as a precursor and examined for its electrocatalytic activity towards glucose without the need for enzymes. The electron microscopy pictures and X‐ray photoelectron spectroscopy (XPS) of Ni‐MOFs/G, with the cyclic voltammetry (CV) curves of the synthesized materials. The Ni_2_P nanoparticles produced from MOFs and the graphene film exhibited remarkable sensitivity to glucose molecules due to their synergistic interaction. The nanoparticles generated from MOFs maintained their electrocatalytic activity towards glucose molecules, whereas the incorporation of graphene enhanced electron transport, hence augmenting conductivity. CuNi/C sheets were synthesized via the electrodeposition of copper nanoparticles onto nickel/carbon sheets obtained from nickel metal–organic frameworks, demonstrating exceptional sensitivity and stability for glucose in blood serum [120]. The porous architecture of Ni/C obtained from the MOFs facilitates swift electron transmission, whereas the inclusion of Cu improves the electrochemical efficacy of glucose detection (Figure 5b). A nonenzymatic sensor utilizing NiCo_2_O_4_ hollow nanocages, generated from metal–organic frameworks (MOFs), was employed as a catalyst for glucose sensing. The addition of Ni and Co in the MOFs‐derived oxide enhances sensing, selectivity, and stability, hence improving catalytic activity. A material produced from MOFs and based on LDHs was utilized as a nonenzymatic sensor for glucose molecules. NiCo LDH was synthesized from MOFs, utilizing the framework as a template, which imparted excellent electrocatalytic characteristics and heightened sensitivity. The synthesized LDHs were integrated with graphene nanoribbons to improve conductivity, while the nanosheets obtained from MOFs augmented the catalytic performance for glucose, yielding glucose sensors with elevated sensitivity and selectivity [127]. The materials generated from MOFs preserve the structural attributes of MOFs while exhibiting improvements in electrical conductivity, stability, and catalytic activity for electrochemical glucose sensing [68]. The transformation of MOFs necessitates meticulous regulation of the reaction conditions to preserve the framework structure, frequently leading to the degradation of the original MOF structure [128].

### 8.3 Detection of Sweat Biomarkers

MOF structures’ greater porosity and larger surface area help concentrate the analyte, which increases the electrochemical signal’s intensity, improves the sensitivity of the sensor, and increases catalytic capacity [127]. Therefore, the production of MOF structures with high conductivity is required for the electrochemical sensors as well as for their use in batteries, fuel cells, and supercapacitors. There are numerous ways to increase the conductivity of MOFs, including pyrolyzing them, creating MOF structures with crystal defects (amorphous MOFs), improving the metal‐centered electronic structure, adding conductive materials to MOF structures, or creating MOF NPs. Maintaining the necessary porosity structure, promoting molecular transport, and widely dispersing the active sites are the three main goals of the pyrolysis of MOFs. The crystalline MOFs can be treated with heat, ball milling, or high pressure to produce amorphous MOFs. By choosing the right metal and ligand precursors and adjusting the synthesis conditions, they might be produced directly [68]. Furthermore, the MOF’s metal centers have the ability to affect the π‐conjugation layer spacing, which improves electron mobility and adds more active sites.

Because of their strong electrocatalytic conductivity, amorphous or low‐dimensional MOFs may soon be utilized to create monolayer‐modified electrodes for the efficient electrochemical detection of sweat biomarkers (Figure 6). However, the conductivity of electrodes treated with a crystalline MOF monolayer is low. Therefore, it is necessary to immobilize additional conductive materials, like NPs or polymers, onto the electrodes modified by the MOF layer. The creation of flexible, wearable, and highly sensitive sweat sensors requires the electrodes modified with MOF NCs to have a high electrical conductivity [128]. For instance, in the past, CNTs were applied as an electroactive coating to MOF NC‐modified electrodes, giving wearable sensors excellent performance for the detection of cortisol and glucose with a lower detection limit. Furthermore, NiMn‐MOF’s ultrathin nanosheets strengthened the electronic structure, increasing the wearable sensor’s electrical conductivity based on MOF NC. Additionally, following doping with lowered rGO, CuNi‐MOF NCs’ electrical conductivity was improved for use in wearable sweat uric acid and dopamine sensing.

**FIGURE 6:**
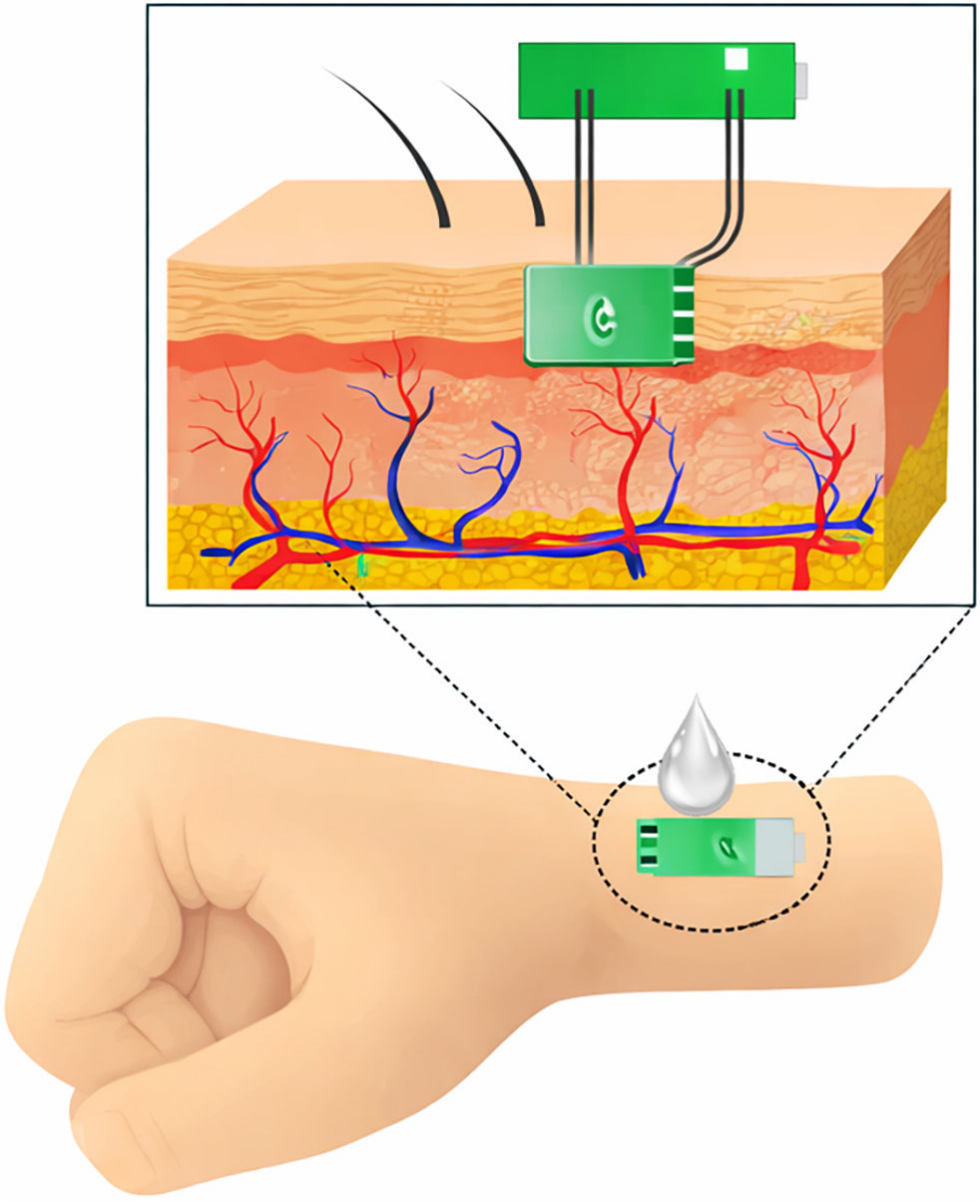
Sweat biomarkers detected by MOFs. Reproduced with permission [128]. Copyright 2024, MDPI.

### 8.4 Detection of Lactate

One essential metabolic product produced by human exertion or metabolic processes is lactate. Blood lactate concentration is kept within a certain range under typical physiological conditions, and it is essential for maintaining energy metabolism, acid–base balance, and other physiological processes. However, when metabolic states are changed as a result of severe exercise, disease impacts, or other circumstances, lactate levels may show aberrant fluctuations. Real‐time lactate monitoring gives athletes and fitness enthusiasts a better knowledge of physical tiredness and exercise intensity, which helps them make informed training plan adjustments, improve athletic performance, and avoid overtraining injuries [129].

Mukundan et al. used a PVA/chitosan multinetwork hydrogel matrix to create a wearable sweat sensor based on bimetallic Fe/Co‐MOF. The multinetwork hydrogel’s enormous surface area and intrinsic better conductivity allowed the sensor to detect L‐lactate in simulated sweat samples with great sensitivity and accuracy. The Fe/Co‐MOF showed a homogeneous, smooth‐surfaced, hexagonal spindle‐like crystal structure [124]. These spikey features improved stability and greatly expanded the material’s specific surface area. The Fe/Co‐MOF hydrogel‐based sweat sensor for L‐lactate uses its oxidation process as its detecting mechanism, as depicted in Figure 7. Because of the active sites and functional groups on the MOF surface, L‐lactate molecules adsorb onto the MOF‐based hydrogel when they are added to the system. The adsorbed L‐lactate molecules oxidize when potential is added. Two protons (H^+^) and two electrons (e^−^) are released when L‐lactate (C_3_H_6_O_3_) is oxidized to pyruvate (C_3_H_4_O_3_) in the chemical reaction. The Fe/Co‐MOF hydrogel catalyzes the electrochemical oxidation of L‐lactate. Sensing is made possible by the correlation between lactate concentration and the current augmentation brought on by electron release.

**FIGURE 7:**
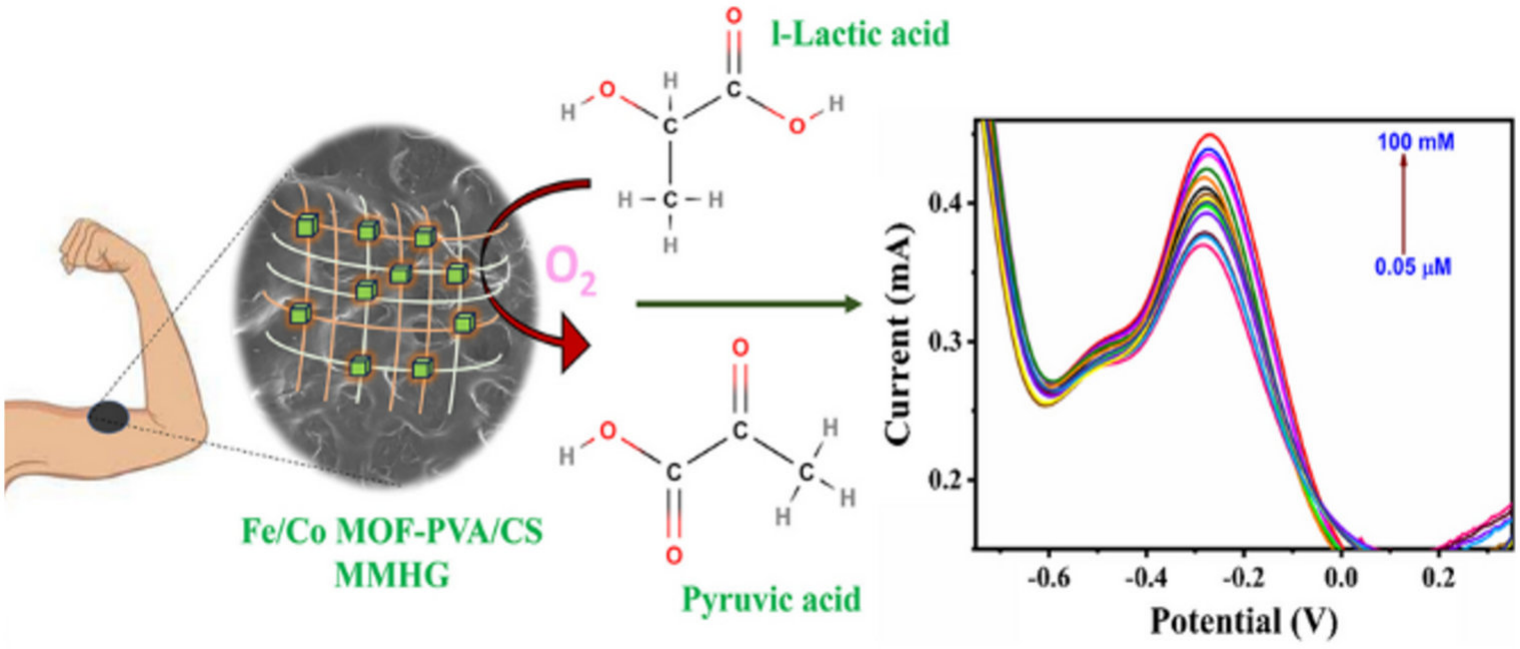
Wearable sweat made of a bimetallic Fe/Co‐(MOF) hydrogel. Reproduced with permission [124]. Copyright 2024, Springer.

### 8.5 Detection of Uric Acid

The growing importance of health monitoring and the quick advancement of electronic device technology have sparked a lot of interest in wearable technology that can be customized to meet the demands of each user. Nevertheless, there are still certain restrictions on the ability of the wearable technology now employed to track health status to provide thorough evaluations [130]. It is more easy, straightforward, and safer to get physiological signals by directly collecting sweat from the skin’s surface since it is an easily executable and direct method that overcomes privacy concerns connected with physical implementation. CuNi‐MOF@rGO, a high‐activity electrocatalyst, was created by Cuncun Wang et al. in 2024, and its electrochemical mechanism and feasibility for concurrently detecting low‐concentration biomarkers UA were examined as depicted in Figure 8. The interaction between the sample and the noncoordinating carboxylate group produces effective separation signals for DA. The wearable biomimetic biosensor has a sensitivity of 0.026 μA μM^−1 ^cm^−2^ for UA and a broad linear range of 10–1000 μM. Its detection limit is 9.09 μM. Therefore, our sensor does a great job of identifying biomarkers at low concentrations. A microfluidic‐controlled apparatus with a hydrophilic alteration in the microchannel to enhance sweat collecting. According to experimental findings, ink flow is best at 2% concentration [121]. All things considered, a novel and extremely active electrocatalyst was created, thereby facilitating the simultaneous detection of low‐concentration UA biomarkers [131, 132].

**FIGURE 8:**
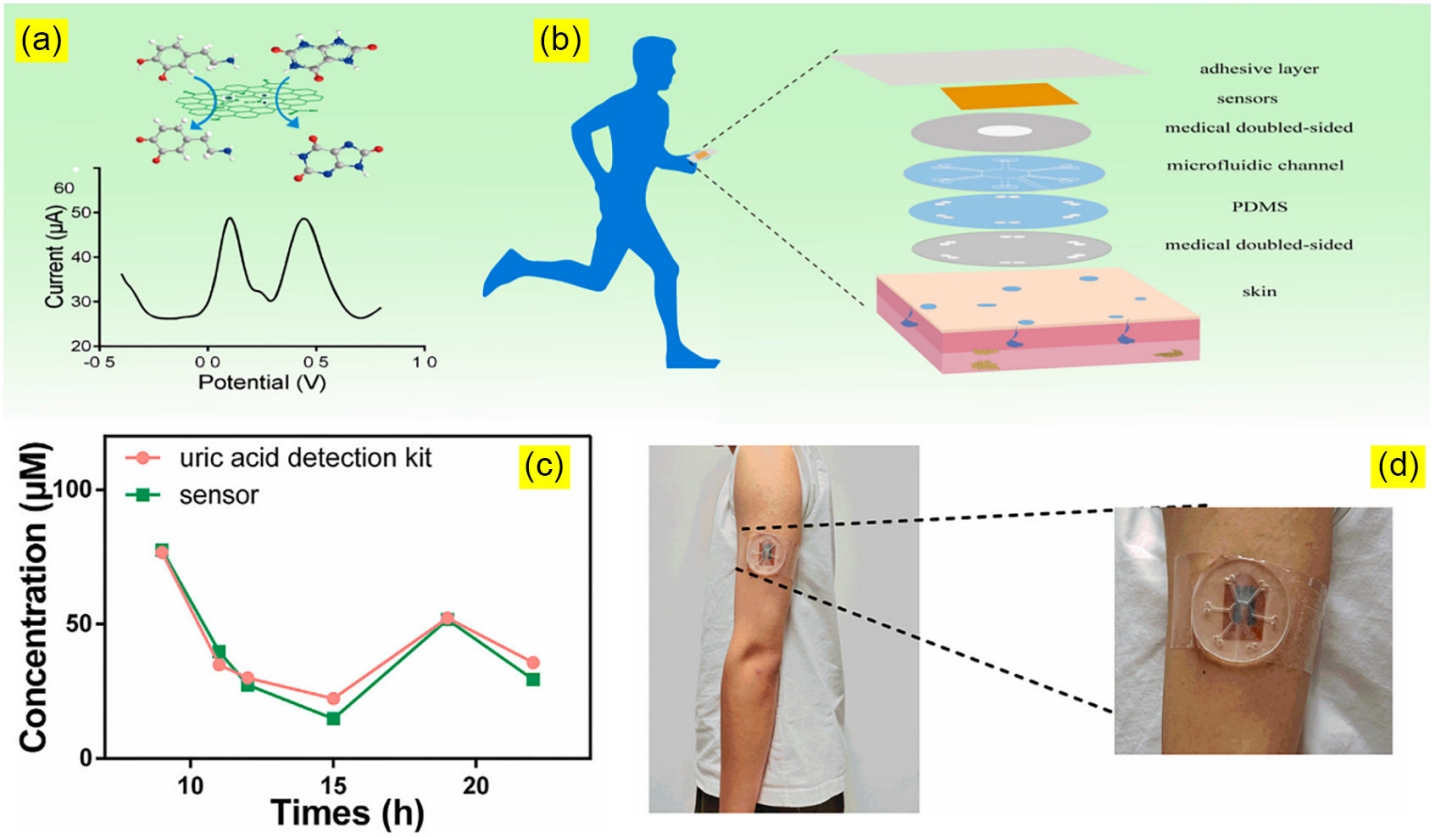
(a) Possible DA and UA electrochemical oxidation processes. (b) Flexible wearable sensor schematic with top cover and layers. (c) Uric acid testing kits. (d) The microfluidic and electrode layers form a sweat microfluidic sensor. Reproduced with permission [121]. Copyright 2024, IOP Publishing.

### 8.6 Detection of Environmental Monitoring

The capability to identify and measure gaseous species with high accuracy is credited to be of great significance in a very wide array of applications, such as environmental surveillance, health and safety of the population, industry‐related process regulation, medical diagnostics, energy usage, and climate change studies, as depicted in Figure 9 [133]. In most practical situations, the occurrence of dangerous or polluting gases even at very low concentrations can pose serious risks to human health and infrastructure [134]. As a result, the development of efficient gas‐sensing technologies has become a major priority [135]. Though sensing devices must satisfy basic analytical requirements such as sensitivity, selectivity, and reproducibility, additional real‐world constraints, including low cost, portability, low power consumption, long‐term stability, and remote data transmission, strongly influence sensor design and material selection as tabulated in Table 2. Consequently, a wide range of gas‐sensing devices have been developed based on different transduction mechanisms and sensing materials [154, 155]. A comparison table of earlier reported MOF based electrochemical sensors for environmental sensing as tabulated in Table 2.

**FIGURE 9:**
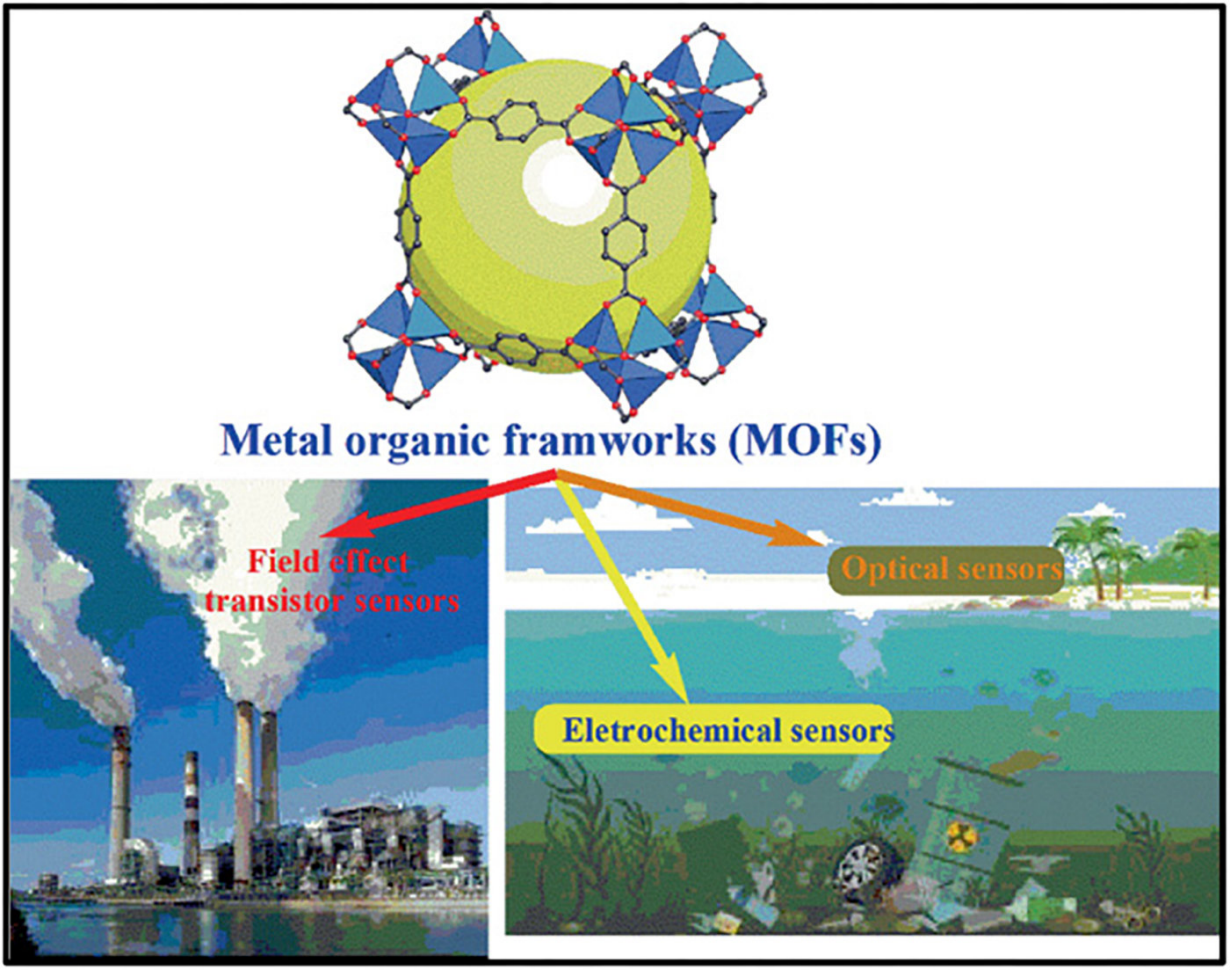
MOFs as Sensors for Environmental Monitoring. Reproduced with permission [133]. Copyright 2022, Springer.

**TABLE 2:** MOFs for Environmental Monitoring Applications.

Analyte	MOF composite	Sensing mechanism	Detection limit/range	Features	References
Pb^2+^, As^3+^, Hg^2+^, Cd^2+^	Fe‐MOF@mFe_3_O_4_@mC nanocomposite	Electrochemical (DPV, aptamer‐based sensing)	LOD: 6.73 pM (As^3+^), Range: 0.01–10 nM	High selectivity, strong host–guest interaction, excellent dispersion	[122, 123]
Pb^2+^ (DNA sensing)	Iron‐porphyrinic MOF	DNA‐functionalized electrochemical sensing	Ultratrace detection	High specificity via biomolecular recognition	[122]
Organophosphorus pesticides (e.g., parathion‐methyl)	Zr‐LMOF (Zr‐based luminescent MOF)	Luminescence quenching/optical sensing	LOD: 0.115 µg kg^−1^; Range: 70 µg kg^−1^–5 mg kg^−1^	Rapid detection, visual sensing, high sensitivity	[136]
OPP residues in food & water	Zr(IV)‐based MOFs	Fluorescence‐based sensing	Trace‐level detection	In situ monitoring, cost‐effective	[136, 137, 138, 139, 140]
NOx, CO, CO_2_, SO_2_, NH_3_, VOCs	MOF‐based FET sensors (MOF channel materials)	Conductance change (chemiresistive/FET)	ppm–ppb level detection	High sensitivity, rapid response, tunable pores	[141, 142, 143, 144]
VOCs	MOF–metal oxide/MOF–carbon composites	Adsorption‐induced signal modulation	Low concentration detection	Enhanced conductivity, stability	[140, 145]
Toxic industrial gases	Ionic liquid–MOF hybrids	Amperometric electrochemical sensing	Real‐time detection	High stability, low power consumption	[146, 147, 148, 149, 150, 151, 152, 153]

### 8.7 Detection of Heavy Metals

Heavy metals like Pb, Cu, As, Cd, Hg, and others that are prevalent in the environment can cause major health issues for people and will never biodegrade in ecological systems [145, 156, 157]. The trace concentrations of heavy metal ions such as Pb(III), Cu(III), As(III), Cd(III), Hg(III), and others would become widely contaminated with persistently harmful contamination due to easy migration of natural activities [158, 159]. Environmental contamination was the most pressing issue of the twenty‐first century. A significant study has been conducted on heavy metal ions, particularly on the experimental procedures and methods used to quantify the pollutants in drinking water. It is evident that the rising concentration of heavy metals, along with other pollutants, has resulted in significant issues with a high environmental impact.

Mesoporous Fe_3_O_4_@C nanocapsules and MOF based on Fe(III) are described. Using the solvothermal approach, mFe_3_O_4_@C nanocapsules were first created by combining SiO_2_ nanoparticles with ferrocene [122]. This was followed by ammonia etching, which produced a core–shell configuration. The Fe‐MOF@mFe_3_O_4_@mC nanocomposite was created by hydrothermally reacting the produced mFe_3_O_4_@C with 2‐amino‐terephthalic acid and FeCl_3_·6H_2_O at 120 °C. The resulting mesoporous MOF‐based nanocomposite has a large surface area, good electrochemical performance, and good dispersion ability. As was previously mentioned, Pb^2+^ was determined using DNA‐functionalized iron‐porphyrinic as a signal probe. In order to determine As^3+^, aptamer strands were immobilized over a Fe‐MOF@mFe_3_O_4_@mC nanocomposite, utilizing the benefits of hydrogen bonding and supramolecular stacking. Furthermore, DPV measurements of the Fe‐MOF/Fe_3_O_4_/mC nanocomposite were performed, and electrochemical efficiency was examined by detecting As^3+^ concentrations ranging from 0.01 to 10.0 nM, with a detection limit of 6.73 pM. Fe‐MOF/Fe_3_O_4_/mC nanocomposite was used to study the selective quantification of arsenic ions in the presence of Ag^+^, Zn^2+^, Ca^2+^, and Mn^2+^ metal ions. It has been noted that even when additional metal ions are present, the electrochemical reaction to the fact that As^3+^ doesn’t change indicates how extremely selective the suggested modified electrode is for detecting arsenic [123]. The nanocomposite electrode demonstrated acceptable selectivity and appropriate repeatability of around 4.66% for the detection of arsenic, as shown in Figure 10.

**FIGURE 10:**
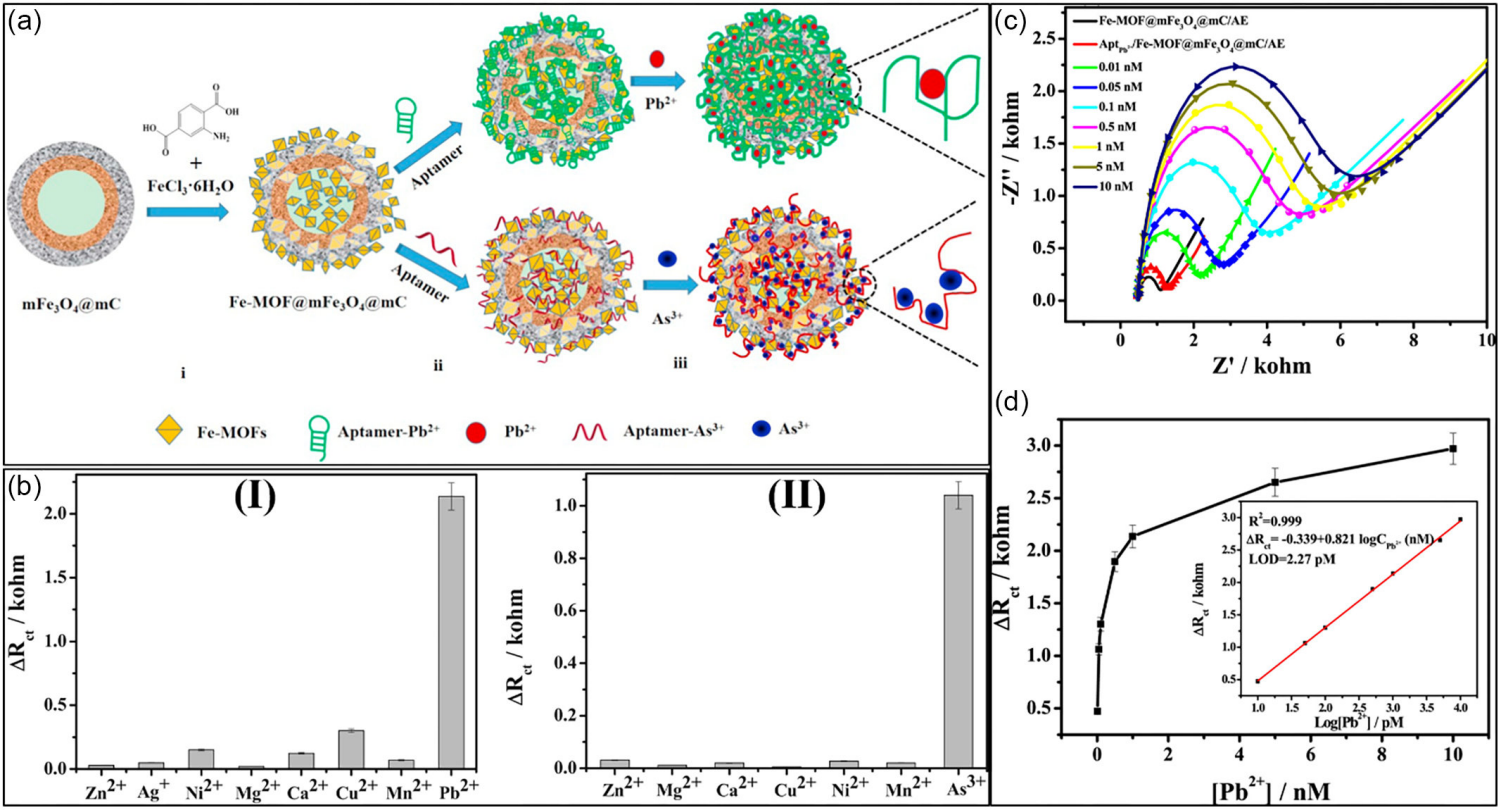
(a) Electrochemical sensing of As^3+^. (b) Selectivity of (I) Pb^2+^ and (II) As^3+^. (c) AptPb^2+^/Fe‐MOF@mFe_3_O_4_@mC/AE EIS responses at varying Pb^2+^ concentrations. (d) The modified electrode’s Δ*R*_ct_ dependence on Pb^2+^ concentration. Reproduced with permission [122]. Copyright 2017, Elsevier.

### 8.8 Detection of Pesticides

Pesticide role in modern agriculture is quite significant as it assists in averting attacked crops, weeds, and diseases to ensure food security among the ever‐growing world population [136, 137, 138]. Organophosphorus pesticides (OPPS) form some of the most popular groups of pesticides because of their great efficiency and relatively economical price. Nonetheless, the extensive and unregulated use of such chemicals has led to the consistent emission of pesticide residues to the environment, causing grave international apprehensions due to the persistence and toxicity of these chemicals [139, 140]. Organophosphorus pesticide residues emitted to the soil, water, and farm products do not easily decompose, which in turn stays in the ecosystem and gets consumed by the food chain, causing long‐term exposure in people and animals, as shown in Figure 11. Many reports have demonstrated that human health can be adversely impacted by being exposed to OPPs, especially by means of inhibiting the activity of acetylcholinesterase in the nervous system, resulting in neurological, respiratory, immune, and endocrine diseases. There is also long‐term exposure, which is linked to mutagenic and carcinogenic effects and acute poisoning leading to fatal consequences [141].

**FIGURE 11:**
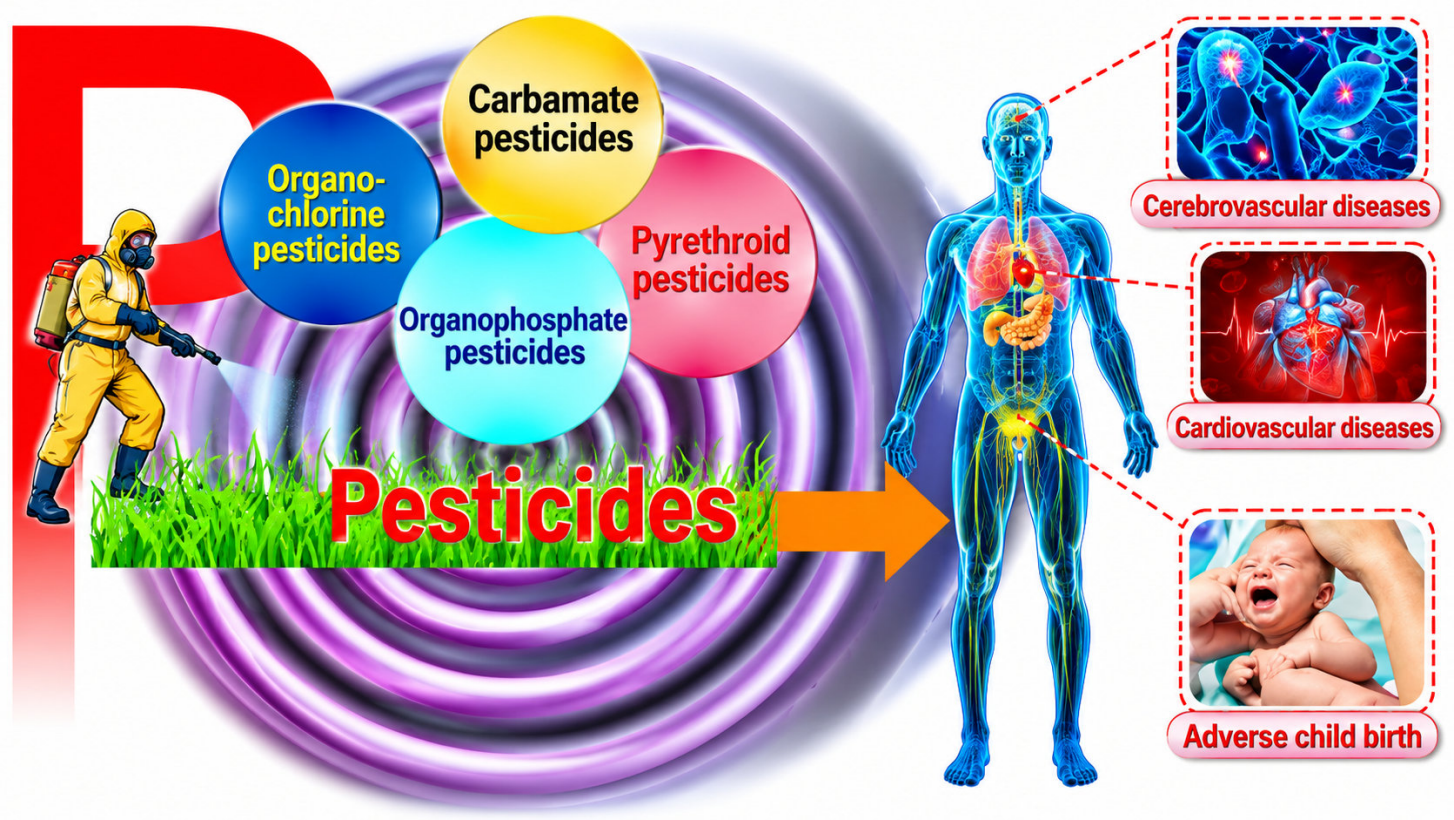
Several widely overused pesticides across the globe pose serious threats to human health. Reproduced with permission [138]. Copyright 2023, Elsevier.

The rapid and inexpensive synthesis of H_2_O‐stable Zr‐LMOF and its application for in situ OPP identification are described in Figure 12 [136]. The Zr‐MOF is made using Zr(IV) and H_4_TCPB. The synthetic Zr‐LMOF acts as an indication of the presence of OPP parathion methyl and absorbs it rapidly. With a high LR of 70 µg kg^−1^–5.0 mg kg^−1^, a low LOD of 0.115 µg kg^−1^ (0.438 nM) could be achieved. Pleasurable retrievals of 78% to 107% were obtained for environmental and dietary samples that were tampered with. Furthermore, without causing any harm, the Zr‐LMOF was utilized to model rapid, in‐place imaging recognition of pesticide residues on fresh yield; after only 5 min, visual signals began to appear under ultraviolet light. These results suggest that the Zr‐LMOF may be able to detect OPP contamination in situ, at low cost, and with a visual signal that is easy to understand.

**FIGURE 12:**
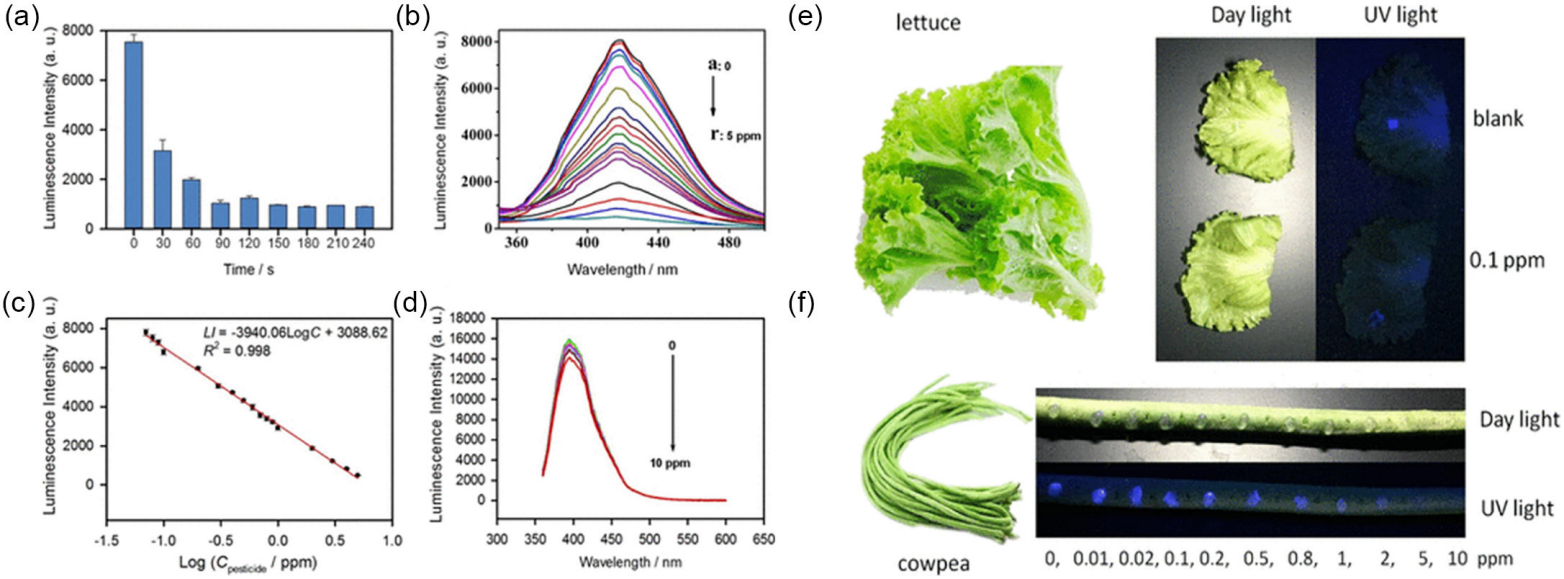
(a) Intensity of luminescence at various reaction periods following the addition of pesticide. (b) Zr‐LMOF luminescence emission spectra at varying parathion‐methyl concentrations. (c) Zr‐LMOF’s maximum luminescence intensity in relation to parathion‐methyl. (d) H_4_TCPB luminosity spectra at various parathion‐methyl concentrations. (e) A 0.1 ppm parathion‐methyl solution was sprayed on the surface of fresh lettuce to mimic pesticide residue; and Parathion‐methyl was rapidly and visibly imaged. (f) To mimic pesticide residue, cowpea was smeared with solutions containing varying parathion‐methyl concentrations. Reproduced with permission [136]. Copyright 2019, ACS.

### 8.9 Detection of Volatile Gas Sensing

Strong host–guest interactions allow for the adsorption of guest molecules due to MOFs’ enormous intrinsic porosities (>90% free volumes), changeable internal surface areas, and high degree of crystallinity. MOFs’ sustaining pores give guest molecules a natural home, which raises the likelihood of guest–host interactions and the sensitivity of the sensing [139]. The strength of analyte binding to the MOF is the primary determinant of sensitivity, although the signal transduction technique also plays a role. Higher reactions are thus the result of stronger binding. MOF‐based materials exhibit great promise as sensing materials for gases as well as water contaminants due to their distinct physical and chemical characteristics [140]. Source and drain electrodes, channel material, gate oxide, and gate electrode make up a FET gas sensor (Figure 13a). The channel material is essential for tracking how the physically adsorbed target gases alter the conductance in the sensor [141]. Thus, the sensing performance is determined by intrinsic properties of the channel material, such as the band gap, carrier mobility, and work function. Depending on the semiconductor type and reducing or oxidizing gas, the conductance in the channel materials may rise or decrease as the gas and channel material interact (Figure 13b). The conductance can change as a result of the adsorbed gas molecules altering the local carrier concentration in the channel material. Due to their fast response to low‐concentration gases and lack of chemical agents, FET sensors have found extensive application in gas detection [142, 144]. In order to enhance the sensing capability, MOF‐based materials have recently been used as the sensing channel in FET sensors or in combination with other gas‐sensitive materials.

**FIGURE 13:**
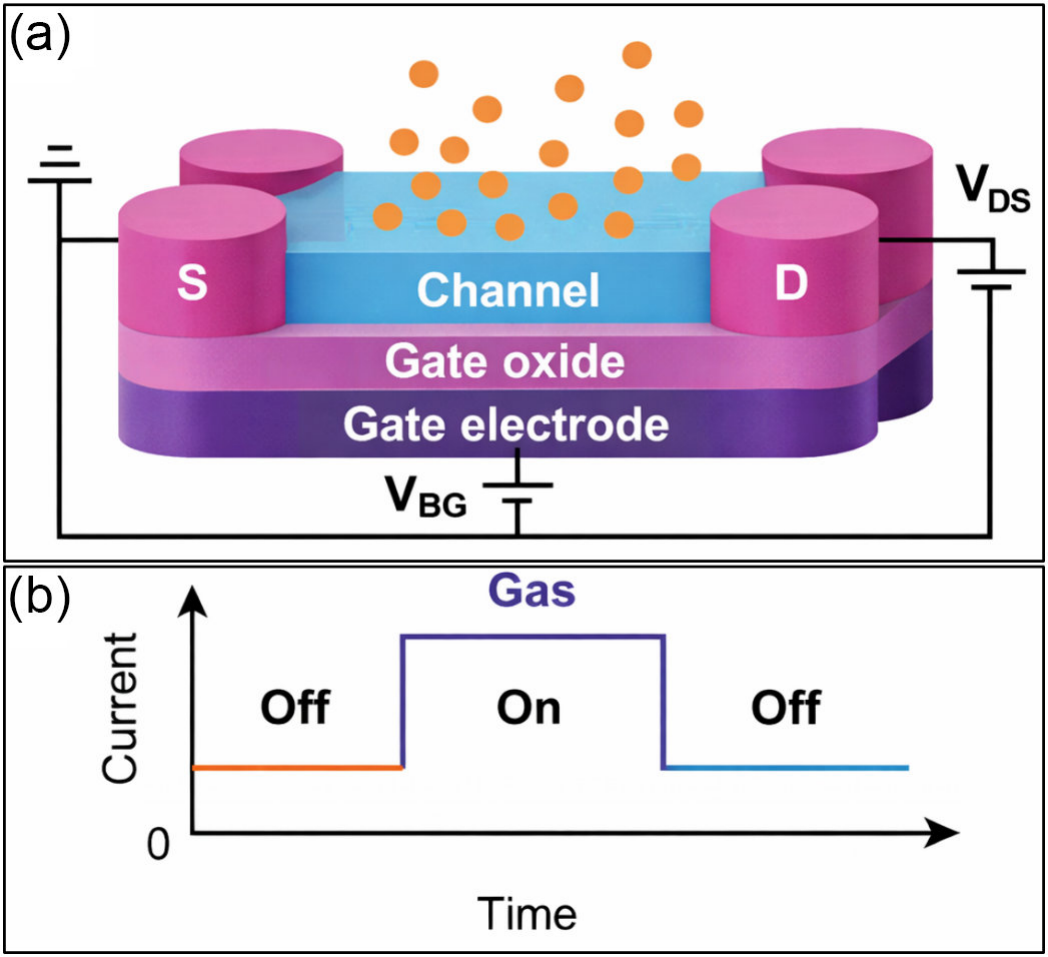
(a) A schematic representation of an FET sensor showing the gate electrode, channel material, source and drain electrodes, and gate oxide. (b) After gas adsorption and desorption, the sensor current changes. Reproduced with permission [141]. Copyright 2017, Royal Society of Chemistry.

Here, the emphasis on ionic liquids (ILs) and metal–organic frameworks (MOFs) as material‐centered systems (either separately or in hybrid form) has become more prominent [146, 147]. Gas sensors find a wide range of applications in the process of environmental pollution control, industrial safety, to detect toxic and flammable gases, and medical diagnostics through the analysis of breath and blood oxygen levels [148, 149, 150, 151]. Some of the typical target gases are nitrogen oxides, carbon monoxide, carbon dioxide, sulfur dioxide, ozone, ammonia, and volatile organic compounds, all of which are extremely hazardous to the environment and human health. Amperometric electrochemical gas sensors are considered as one of the most popular platforms because of their low cost, sensitivity, low power consummation, and minimization capabilities [152]. The use of ionic liquids to replace water as an electrolyte has enhanced the stability, lifetime, and performance of these sensors in hard or dry operating environments [153].

## 9 Challenges & Future Perspectives

Although MOF‐based and MOF‐derived electrochemical sensing platforms have been developed quickly, there are still challenges before the performance of these platforms in the lab can be extended to become reliable electrochemical sensing platforms in the real world. However, the challenges are not only to obtain low detection limits but also to accurately and repeatably synthesize the material, control the structure–property relationship, ensure long‐term stability, achieve selectivity in complex matrices, and develop robust electrode preparation and integration with practical sensing systems [160]. The difficulty of reproducing MOF synthesis and then converting them to functional sensing materials is another major hurdle. The MOF properties depend on the purity of the precursors, the ratio of metal to the ligand, the composition of the solvent, the pH, the concentration of the modulator, the temperature of the reaction, the time of the reaction, and the crystallization conditions.

MOF‐based materials may also be influenced by other factors like the calcination temperature, heating rate, treatment time, and the atmosphere used to treat the material, which can affect the morphology, porosity, graphitization, and chemical state of the metals [161]. Thus, different electrochemical properties can be observed in two materials with the same MOF‐derived composition. Future reports need to therefore include detailed synthesis and post‐treatment parameters, if possible, and standardization of the procedure of characterization in order to be able to compare the analytical performances of the studies. The enhanced conductivity of many MOFs compared to their pristine counterparts is one advantage of MOFs derived from MOFs, but a higher conductivity does not necessarily mean better sensing ability. Greater graphitization or even the addition of metallic phases could improve electron transport properties but can also change the surface chemistry, pore accessibility and number and density of active sites. Likewise, too great a thermal conversion can lead to the collapse of pores or aggregation of metal containing phases [162]. Conductivity, porosity, active site accessibility, and structural stability should, therefore, be controlled instead of optimizing the value of a single parameter in future research. Simple buffer solutions give high sensitivities and low detection limits but not necessarily for practical use in sensors. Proteins, salts, metabolites, organic compounds, and competing ions are found in biological and environmental samples that may affect the diffusion process, adsorption, electron transfer, or catalytic reactions of the analytes [163].

Pristine MOFs could also suffer from loss of well‐defined pore‐seeing effect in their transformation. Thus, future sensing platforms should be integrated with MOF‐based conductive/catalytic architecture and also have selective recognition elements like aptamers, enzymes, antibodies, molecularly imprinted polymers, or suitably functionalized surfaces. A complex‐matrix test should be more important for the assessment of the sensing platform than a buffer‐based interference test and should be authentic or realistically simulated. Another significant constraint is the scalability of the synthesis of MOF and the fabrication of the electrode. Synthetic methods of MOFs reported are often complicated, use expensive organic ligands and organic solvents, or need multistep processing or energy intensive thermal conversion. These techniques yield good materials on the lab scale, but their economic and environmental sustainability may decline on large scale production [164]. The synthesis of this material using less expensive precursors, simpler synthetic pathways, aqueous or environmentally friendly solvents, lower energy conversion strategy, and scalable deposition should be explored in the future. A comprehensive economic evaluation of the process should be performed from the precursor synthesis, purification, conversion, electrode fabrication, and waste generation and not only the production cost of the sensing material. Coordinated integration of flexible substrates, miniaturized electronics, microfluidic systems, and wireless communication platforms are increasingly essential in the development of practical electrochemical sensors. The structure, surface chemistry, and composition of MOF‐derived materials can be designed for specific interfaces, which makes them suitable for this purpose [165]. While a material that works well on a standard electrode can work well on a flexible material, it does not necessarily translate to the same performance when the material is repeatedly subjected to mechanical deformation or when deposited on a flexible material. Adhesion of electrodes, mechanical durability, exposure of the electrolytes, bending stability, and retention of the signals over time should thus be considered in future studies. Combination of MOF‐derived sensing layers with conductive polymers, carbon nanomaterials, flexible electrodes, and microfluidic sampling systems can offer a path forward towards compact and wearable sensing platforms [166]. We believe that the focus of MOF based electrochemical sensing should shift from material performance optimization to system level engineering. Future studies should quantify the relationship between the synthesis conditions, structural features, electrochemical properties, and analytical performance, instead of reporting only sensitivity and detection limits. A combination of advanced characterization, computational modeling, and data‐based approaches would enable to identify the structural parameters that give better sensing capabilities and avoid empirical trial and error optimization. A future direction that is also promising is the creation of multifunctional interfaces based on MOFs with different components having complementary functions. A porous carbon phase can offer conductivity, metal‐containing sites can provide electrocatalytic activity, and an aptamer or molecularly imprinted layer can provide molecular recognition, for instance. Hierarchical structures can offer improved conductivity, catalytic activity, selectivity, and stability in a single step, rather than by employing a single material component [167]. In general, the field would benefit from more standardization of testing methods, more use of authentic samples, more attention to long‐term operation, more concern for scalable fabrication, and integration with devices. The ability of MOF‐based electrochemical sensors to deliver impressive analytical performance will depend on these factors, which will enable the development of reliable sensors for clinical diagnostics, environmental monitoring, food analysis, and wearable health monitoring.

## 10 Conclusion

The structural tunability, large surface area, chemical functionality, and capacity for the synthesis of conductive and catalytically active derivatives of MOFs have made them promising platforms for electrochemical sensing, offering a variety of opportunities for engineering sensing interfaces. Several limitations of pristine MOFs can be addressed by converting MOFs to carbonaceous materials, metal/metal oxide phases, and hybrid structures, as has been discussed throughout this review, especially their relatively poor electrical conductivity and electrochemical instability. The scaling up from laboratory demonstrations to reliable practical sensing platforms however is still challenging. The reproducibility and scalability of the MOF synthesis and MOF‐derived material synthesis are one of the key issues. The MOF properties can be highly sensitive to the purity of the precursors, the ratio of metals to the ligands used, the composition of the solvents used, reaction temperature, reaction time, and the conditions of post‐treatment. Besides the above variables, the temperature of the calcination, heating rate, atmosphere, and treatment time are also important factors that can significantly affect carbonization, porosity, dispersion of metals, and the oxidation state during thermal conversion. Such changes in these parameters may, in turn, lead to differences in the active‐site density, conductivity, pore structure, and electrochemical response. Thus, standardized synthesis procedures, tighter control over the variation of the batches, and scalable processing procedures must be provided to assure reliable manufacturing of sensors. The economic issues of MOF‐based sensing materials also need to be paid more attention. While MOFs have many structural and chemical benefits, many of the frameworks that have been studied as precursors use ligands, metal salts, solvents, or multistep synthesis procedures which are relatively costly. Bench‐scale production of the sensors thus necessitates a careful consideration on the cost of the precursors, consumption of solvents, energy needed for thermal conversion, purification processes, and yield of the material. The use of cheaper metal sources, easier synthetic pathways, less environmental friendly solvents, renewable or biomass based ligands, and less energy‐consuming conversion processes should be investigated more and more in future research.

## Author Contributions

**Nithish Kumar M** **:** conceptualization, methodology, investigation, data curation, writing – original draft. **Prabu S** **:** methodology, validation, formal analysis, writing – review & editing. **Munusamy Settu** **:** investigation, resources, data curation. **P. Saravanan** **:** project administration. **Vinothkumar J** **:** software, visualization, data curation. **S. Rajkumar** **:** conceptualization, supervision, project administration, writing – review & editing. **Krishna** **Prakash Arunachalam** **:** investigation, resources. **Sandeep Eswaran Panchu:** validation, formal analysis, writing – review & editing. **Hendrik C. Swart:** validation, formal analysis, writing – review & editing.

## Conflicts of Interest

The authors declare no conflicts of interest.

## Data Availability

Data will be made available on request.

## References

[1] A. Yousaf , Z. Zhao , and M. W. Boota , “Enhancing Electrochemical Detection With Metal‐Organic Frameworks: Advances in Functionalization, Synthesis, and Application,” Journal of Solid State Electrochemistry 30 (2026): 1009–1032, 10.1007/s10008-025-06477-w.

[2] A. Boakye , K. Yu , H. Chai , et al., “Two‐Dimensional Nickel Porphyrinic Metal–Organic Framework‐Modified Electrode for Electrochemical Sensing,” Langmuir 40, no. 5 (2024): 2708–2718, 10.1021/acs.langmuir.3c03257. 38277771

[3] Q. Wu , J. Liang , D. Wang , R. Wang , and C. Janiak , “Host Molecules Inside Metal–Organic Frameworks: Host@MOF and Guest@host@MOF (Matrjoschka) Materials,” Chemical Society Reviews 54 (2025): 601–622, 10.1039/D4CS00371C. 39589788

[4] A. Felix Sahayaraj , H. Joy Prabu , J. Maniraj , et al., “Metal–Organic Frameworks (MOFs): The Next Generation of Materials for Catalysis, Gas Storage, and Separation,” Journal of Inorganic and Organometallic Polymers and Materials 33 (2023): 1757–1781, 10.1007/s10904-023-02657-1.

[5] D. Liu , K. Lu , C. Poon , and W. Lin , “Metal–Organic Frameworks as Sensory Materials and Imaging Agents,” Inorganic Chemistry 53 (2014): 1916–1924, 10.1021/ic402194c. PMC4108452 24251853

[6] H. Karimi‐Maleh , F. Karimi , M. Alizadeh , and A. L. Sanati , “Electrochemical Sensors, a Bright Future in the Fabrication of Portable Kits in Analytical Systems,” Chemical Record 20 (2020): 682–692, 10.1002/tcr.v20.7. 31845511

[7] G. Xu , C. Zhu , and G. Gao , “Recent Progress of Advanced Conductive Metal–Organic Frameworks: Precise Synthesis, Electrochemical Energy Storage Applications, and Future Challenges,” Small 18 (2022): 2203140, 10.1002/smll.v18.44. 36050887

[8] H. Chai , X. Sun , X. Tan , et al., “Electrochemical Gating of d‐Band Engineering in Hierarchically Bridged Dual‐Site Nanozymes for Synergistic Cascade Catalysis and Wearable Biosensing,” Analytical Chemistry 98, no. 5 (2026): 3919–3933, 10.1021/acs.analchem.5c06201. 41622850

[9] X. Zhang , A. Chen , M. Zhong , et al., “Metal–Organic Frameworks (MOFs) and MOF‐Derived Materials for Energy Storage and Conversion,” Electrochemical Energy Reviews 2 (2019): 29–104, 10.1007/s41918-018-0024-x.

[10] R. A. Joghani , A. A. Rafati , J. Ghodsi , A. Zolfaghari Asl , and A. Feizollahi , “A Simple and Fast Epinephrine Voltammetry Sensor Using Reduced Graphene Oxide and Iron Metal–Organic Framework,” Journal of Solid State Electrochemistry 27 (2023): 3347–3361, 10.1007/s10008-023-05597-5.

[11] O. M. Yaghi , H. Li , C. Davis , D. Richardson , and T. L. Groy , “Synthetic Strategies, Structure Patterns, and Emerging Properties in the Chemistry of Modular Porous Solids,” Accounts of Chemical Research 31 (1998): 474–484, 10.1021/ar970151f.

[12] L. T. Zhang , Y. Zhou , and S. T. Han , “The Role of Metal–Organic Frameworks in Electronic Sensors,” Angewandte Chemie International Edition 60 (2021): 15320–15340. 10.1002/anie.202006402 32845072

[13] B. Olaniyan and B. Saha , “Comparison of Catalytic Activity of ZIF‐8 and Zr/ZIF‐8 for Greener Synthesis of Chloromethyl Ethylene Carbonate by CO_2_ Utilization,” Energies 13 (2020): 521, 10.3390/en13030521.

[14] K. Yan , P. Karthick Kannan , D. Doonyapisut , K. Wu , C. H. Chung , and J. Zhang , “Advanced Functional Electroactive and Photoactive Materials for Monitoring the Environmental Pollutants,” Advanced Functional Materials 31 (2021): 2008227.

[15] B. Yeskendir , J. P. Dacquin , Y. Lorgouilloux , C. Courtois , S. Royer , and J. Dhainaut , “From Metal–Organic Framework Powders to Shaped Solids: Recent Developments and Challenges,” Materials Advances 2 (2021): 7139–7186, 10.1039/D1MA00630D.

[16] I. Mirkovic , Factors Affecting the Morphology and Composition of MOF Crystals (Doctoral dissertation, 2020).

[17] S. Yuan , L. Feng , K. Wang , et al., “Stable Metal–Organic Frameworks: Design, Synthesis, and Applications,” Advanced Materials (Deerfield Beach, Fla.) 30 (2018): 1704303. 10.1002/adma.201704303 29430732

[18] Y. Fan , C. Long , Y. Cai , Y. Hu , and L. Peng , “Functionalized Metal‐Organic Framework and MOF‐Derived Materials for Bone Regeneration Applications,” Frontiers in Bioengineering and Biotechnology 13 (2025): 1645657, 10.3389/fbioe.2025.1645657. PMC12425977 40948972

[19] Y. Shen , A. Tissot , and C. Serre , “Recent Progress on MOF‐Based Optical Sensors for VOC Sensing,” Chemical Science 13 (2022): 13978–14007, 10.1039/D2SC04314A. PMC9728564 36540831

[20] J. Duan , Y. Pan , G. Liu , and W. Jin , “Metal‐Organic Framework Adsorbents and Membranes for Separation Applications,” Current Opinion in Chemical Engineering 20 (2018): 122–131, 10.1016/j.coche.2018.04.005.

[21] T. Surek , “Crystal Growth and Materials Research in Photovoltaics: Progress and Challenges,” Journal of Crystal Growth 275 (2005): 292–304, 10.1016/j.jcrysgro.2004.10.093.

[22] A. W. Vere , Crystal Growth: Principles and Progress (Springer, 2013).

[23] L. Ndlwana , N. Raleie , K. M. Dimpe , et al., “Sustainable Hydrothermal and Solvothermal Synthesis of Advanced Carbon Materials in Multidimensional Applications: A Review,” Materials 14 (2021): 5094, 10.3390/ma14175094. PMC8434334 34501183

[24] R. H. Zee , Z. Xiao , B. A. Chin , and J. Liu , “Processing of Single Crystals for High Temperature Applications,” Journal of Materials Processing Technology 113 (2001): 75–80, 10.1016/S0924-0136(01)00588-X.

[25] R. I. Walton , “Subcritical Solvothermal Synthesis of Condensed Inorganic Materials,” Chemical Society Reviews 31 (2002): 230–238, 10.1039/b105762f. 12164069

[26] J. J. De Yoreo and P. G. Vekilov , “Principles of Crystal Nucleation and Growth,” Reviews in Mineralogy and Geochemistry 54 (2003): 57–93, 10.2113/0540057.

[27] M. Kalaj and S. M. Cohen , “Postsynthetic Modification: An Enabling Technology for the Advancement of Metal–Organic Frameworks,” ACS Central Science 6 (2020): 1046–1057, 10.1021/acscentsci.0c00690. PMC7379093 32724840

[28] J. Ferrando‐Soria , H. Khajavi , P. Serra‐Crespo , et al., “Highly Selective Chemical Sensing in a Luminescent Nanoporous Magnet,” Advanced Materials (Deerfield Beach, Fla.) 24 (2012): 5625–5629, 10.1002/adma.v24.41. 22887721

[29] L. N. Jin , Q. Liu , and W. Y. Sun , “An Introduction to Synthesis and Application of Nanoscale Metal–Carboxylate Coordination Polymers,” CrystEngComm 16 (2014): 3816–3828, 10.1039/c3ce41962b.

[30] J. Jacobsen , A. Ienco , R. D’Amato , F. Costantino , and N. Stock , “The Chemistry of Ce‐Based Metal–Organic Frameworks,” Dalton Transactions (Cambridge, England: 2003) 49 (2020): 16551–16586, 10.1039/D0DT02813D. 33146175

[31] J. Yang , A. Sudik , C. Wolverton , and D. J. Siegel , “High Capacity Hydrogen Storage Materials: Attributes for Automotive Applications and Techniques for Materials Discovery,” Chemical Society Reviews 39 (2010): 656–675, 10.1039/B802882F. 20111786

[32] H. J. Scheel , “Historical Aspects of Crystal Growth Technology,” Journal of Crystal Growth 211 (2000): 1–12, 10.1016/S0022-0248(99)00780-0.

[33] D. R. Modeshia and R. I. Walton , “Solvothermal Synthesis of Perovskites and Pyrochlores: Crystallisation of Functional Oxides Under Mild Conditions,” Chemical Society Reviews 39 (2010): 4303–4325, 10.1039/b904702f. 20532260

[34] G. Chen , Y. Peng , G. Zheng , et al., “Polysynthetic Twinned TiAl Single Crystals for High‐Temperature Applications,” Nature Materials 15 (2016): 876–881, 10.1038/nmat4677. 27322822

[35] R. Gani , C. Jiménez‐González , and D. J. Constable , “Method for Selection of Solvents for Promotion of Organic Reactions,” Computers & Chemical Engineering 29 (2005): 1661–1676, 10.1016/j.compchemeng.2005.02.021.

[36] A. R. Katritzky , D. A. Nichols , M. Siskin , R. Murugan , and M. Balasubramanian , “Reactions in High‐Temperature Aqueous Media,” Chemical Reviews 101 (2001): 837–892, 10.1021/cr960103t. 11709860

[37] P. Wu , M. Guo , R. W. Zhang , Q. Huang , G. Wang , and Y. Q. Lan , “When Microplastics/Plastics Meet Metal–Organic Frameworks: Turning Threats Into Opportunities,” Chemical Science 15 (2024): 17781–17798, 10.1039/D4SC05205F. PMC11474910 39421205

[38] T. R. Cook , Y. R. Zheng , and P. J. Stang , “Metal–Organic Frameworks and Self‐Assembled Supramolecular Coordination Complexes: Comparing and Contrasting the Design, Synthesis, and Functionality of Metal–Organic Materials,” Chemical Reviews 113 (2013): 734–777, 10.1021/cr3002824. PMC3764682 23121121

[39] G. Zhang , X. Li , Y. Liu , G. Du , and H. Pang , “Metal–Organic Framework Derived Micro‐/Nano‐Materials: Precise Synthesis and Clean Energy Applications,” Inorganic Chemistry Frontiers 11 (2024): 6275–6306, 10.1039/D4QI01732C.

[40] M. Kim , Y. Lee , and H. R. Moon , “Carboxylate‐Based Metal–Organic Framework and Coordination Polymer Glasses: Progress and Perspectives,” Accounts of Chemical Research 57 (2024): 2347–2357, 10.1021/acs.accounts.4c00290. 39120104

[41] M. Zeeshan and M. Shahid , “State of the Art Developments and Prospects of Metal–Organic Frameworks for Energy Applications,” Dalton Transactions (Cambridge, England: 2003) 51 (2022): 1675–1723, 10.1039/D1DT03113A. 34919099

[42] A. Schneemann , V. Bon , I. Schwedler , I. Senkovska , S. Kaskel , and R. A. Fischer , “Flexible Metal–Organic Frameworks,” Chemical Society Reviews 43 (2014): 6062–6096, 10.1039/C4CS00101J. 24875583

[43] N. Stock and S. Biswas , “Synthesis of Metal‐Organic Frameworks (MOFs): Routes to Various MOF Topologies, Morphologies, and Composites,” Chemical Reviews 112 (2012): 933–969, 10.1021/cr200304e. 22098087

[44] X. Y. Yang , L. H. Chen , Y. Li , J. C. Rooke , C. Sanchez , and B. L. Su , “Hierarchically Porous Materials: Synthesis Strategies and Structure Design,” Chemical Society Reviews 46 (2017): 481–558, 10.1039/C6CS00829A. 27906387

[45] Y. Qian , F. Zhang , and H. Pang , “A Review of MOFs and Their Composites‐Based Photocatalysts: Synthesis and Applications,” Advanced Functional Materials 31 (2021): 2104231, 10.1002/adfm.v31.37.

[46] H. Wang , Q. L. Zhu , R. Zou , and Q. Xu , “Metal‐Organic Frameworks for Energy Applications,” Chem 2 (2017): 52–80, 10.1016/j.chempr.2016.12.002.

[47] H. S. Wang , Y. H. Wang , and Y. Ding , “Development of Biological Metal–Organic Frameworks Designed for Biomedical Applications: From Bio‐Sensing/Bio‐Imaging to Disease Treatment,” Nanoscale Advances 2 (2020): 3788–3797, 10.1039/D0NA00557F. PMC9418943 36132764

[48] S. Kothawade and P. Shende , “Coordination Bonded Stimuli‐Responsive Drug Delivery System of Chemical Actives With Metal in Pharmaceutical Applications,” Coordination Chemistry Reviews 510 (2024): 215851, 10.1016/j.ccr.2024.215851.

[49] L. Du , W. Chen , P. Zhu , Y. Tian , Y. Chen , and C. Wu , “Applications of Functional Metal–Organic Frameworks in Biosensors,” Biotechnology Journal 16 (2021): 1900424, 10.1002/biot.v16.2. 32271998

[50] A. Wang , M. Walden , R. Ettlinger , et al., “Biomedical Metal–Organic Framework Materials: Perspectives and Challenges,” Advanced Functional Materials 34 (2024): 2308589, 10.1002/adfm.v34.43. PMC7617264 39726715

[51] T. Qiu , Z. Liang , W. Guo , H. Tabassum , S. Gao , and R. Zou , “Metal–Organic Framework‐Based Materials for Energy Conversion and Storage,” ACS Energy Letters 5 (2020): 520–532, 10.1021/acsenergylett.9b02625.

[52] S. Roy , Z. Huang , A. Bhunia , et al., “Electrocatalytic Hydrogen Evolution From a Cobaloxime‐Based Metal–Organic Framework Thin Film,” Journal of the American Chemical Society 141 (2019): 15942–15950, 10.1021/jacs.9b07084. PMC6803166 31508946

[53] M. Qiu , H. Wu , L. Cao , et al., “Metal–Organic Nanogel With Sulfonated Three‐Dimensional Continuous Channels as a Proton Conductor,” ACS Applied Materials & Interfaces 12 (2020): 19788–19796, 10.1021/acsami.0c02048. 32264674

[54] R. B. Lin , S. Xiang , W. Zhou , and B. Chen , “Microporous Metal‐Organic Framework Materials for Gas Separation,” Chem 6 (2020): 337–363, 10.1016/j.chempr.2019.10.012.

[55] H. B. Wu and X. W. Lou , “Metal‐Organic Frameworks and Their Derived Materials for Electrochemical Energy Storage and Conversion: Promises and Challenges,” Science Advances 3 (2017): eaap9252, 10.1126/sciadv.aap9252. PMC5714063 29214220

[56] B. Chen , S. Xiang , and G. Qian , “Metal–Organic Frameworks With Functional Pores for Recognition of Small Molecules,” Accounts of Chemical Research 43 (2010): 1115–1124, 10.1021/ar100023y. 20450174

[57] R. Ma , Y. Hou , W. Zhang , Y. T. Pan , S. Huo , and C. Shi , “MOF‐Based Nanocomposites in Polymer Matrix: Progress and Prospects,” Advanced Composites and Hybrid Materials 8 (2025): 351, 10.1007/s42114-025-01435-6.

[58] Y. Xu , S. Zheng , H. Xue , and H. Pang , “Metal–Organic Framework Composites and Their Electrochemical Applications,” Journal of Materials Chemistry A 7 (2019): 7301–7327, 10.1039/C8TA12178H.

[59] Ü. Anika , S. Timur , and Z. Dursun , “Metal Organic Frameworks in Electrochemical and Optical Sensing Platforms: A Review,” Microchim Acta 186 (2019): 196, 10.1007/s00604-019-3321-0. 30788595

[60] J. M. Gonzales , P. R. Martins , D. P. Rocha , et al., “Recent Trends and Perspectives in Electrochemical Sensors Based on MOF‐Derived Materials,” Journal of Materials Chemistry C 9 (2021): 8718–8745, 10.1039/D1TC02025K.

[61] Y. Li , Y. Xu , W. Yang , W. Shen , H. Xue , and H. Pang , “MOF‐Derived Metal Oxide Composites for Advanced Electrochemical Energy Storage,” Small 14 (2018): 1704435, 10.1002/smll.v14.25. 29750438

[62] J. Zhao , Y. Kan , Z. Chen , H. Li , and W. Zhang , “MOFs‐Modified Electrochemical Sensors and the Application in the Detection of Opioids,” Biosensors 13 (2023): 284, 10.3390/bios13020284. PMC9954106 36832051

[63] J. Calbo , M. J. Golomb , and A. Walsh , “Redox‐Active Metal–Organic Frameworks for Energy Conversion and Storage,” Journal of Materials Chemistry A 7, no. 28 (2019): 16571–16597, 10.1039/C9TA04680A.

[64] M. Rezki , M. M. Hossain , T. K. Savage , Y. Tokunou , and S. Tsujimura , “Rational Design of Redox Active Metal Organic Frameworks for Mediated Electron Transfer of Enzymes,” Materials Horizons 12, no. 3 (2025): 760–769, 10.1039/D4MH01538J. 39792379

[65] X. Zhou , X. Chen , B. Yang , et al., “Advancements in Functionalizable Metal–Organic Frameworks for Flexible Sensing Electronics,” Advanced Functional Materials. (2025): 2501683, 10.1002/adfm.v35.32.

[66] V. Gargiulo , M. Alfè , L. Giordano , and S. Lettieri , “Materials for Chemical Sensing: A Comprehensive Review on the Recent Advances and Outlook Using Ionic Liquids, Metal–Organic Frameworks (MOFs), and MOF‐Based Composites,” Chemosensors 10 (2022): 290, 10.3390/chemosensors10080290.

[67] A. L. Moore and L. Shi , “Emerging Challenges and Materials for Thermal Management of Electronics,” Materials Today 17 (2014): 163–174, 10.1016/j.mattod.2014.04.003.

[68] R. Akai , H. Kasai , and K. Oka , “Redox‐Active Metal–Organic Frameworks as Electrode‐Active Materials in Aqueous Rechargeable Batteries,” ACS Applied Electronic Materials 8 (2026): 5763–5772, 10.1021/acsaelm.6c00641.

[69] I. Jeerapan and S. Poorahong , “Flexible and Stretchable Electrochemical Sensing Systems: Materials, Energy Sources, and Integrations,” Journal of the Electrochemical Society 167 (2020): 037573, 10.1149/1945-7111/ab7117.

[70] Z. Y. Gu , C. X. Yang , N. A. Chang , and X. P. Yan , “Metal–Organic Frameworks for Analytical Chemistry: From Sample Collection to Chromatographic Separation,” Accounts of Chemical Research 45 (2012): 734–745, 10.1021/ar2002599. 22404189

[71] M. Z. Hasan , T. T. Dipti , L. Liu , C. Wan , L. Feng , and Z. Yang , “Coating Metal–Organic Frameworks (MOFs) and Associated Composites on Electrodes, Thin Film Polymeric Materials, and Glass Surfaces,” Nanomaterials 15 (2025): 1187, 10.3390/nano15151187. PMC12348584 40801725

[72] S. Zhang , M. Wang , X. Wang , J. Song , and X. Yang , “Electrocatalysis in MOF Films for Flexible Electrochemical Sensing: A Comprehensive Review,” Biosensors 14 (2024): 420, 10.3390/bios14090420. PMC11430114 39329795

[73] S. Chongdar , S. Bhattacharjee , P. Bhanja , and A. Bhaumik , “Porous Organic–Inorganic Hybrid Materials for Catalysis, Energy and Environmental Applications,” Chemical Communications 58 (2022): 3429–3460, 10.1039/D1CC06340E. 35234753

[74] S. Natarajan and P. Mahata , “Metal–Organic Framework Structures–How Closely Are They Related to Classical Inorganic Structures,” Chemical Society Reviews 38 (2009): 2304–2318, 10.1039/b815106g. 19623352

[75] L. Liu , Y. Zhou , S. Liu , and M. Xu , “The Applications of Metal–Organic Frameworks in Electrochemical Sensors,” ChemElectroChem 5 (2018): 6–19, 10.1002/celc.v5.1.

[76] B. Wolfrum , E. Kätelhön , A. Yakushenko , et al., “Nanoscale Electrochemical Sensor Arrays: Redox Cycling Amplification in Dual‐Electrode Systems,” Accounts of Chemical Research 49 (2016): 2031–2040, 10.1021/acs.accounts.6b00333. 27602780

[77] K. S. Devi , M. Rezki , and S. Tsujimura , “Emerging Electrochemical Biosensing Strategies Using Enzyme‐Incorporated Metal‐Organic Frameworks,” Talanta Open 8 (2023): 100263, 10.1016/j.talo.2023.100263.

[78] A. Ji , X. Lu , Z. Du , Y. Zhang , J. Xie , and Z. Ding , “Dual‐Signal MOF Nanozyme Microneedle Patch for on‐Site Monitoring of Hydrogen Peroxide in Postharvest Lettuce,” Biosensors and Bioelectronics. (2026): 118933, 10.1016/j.bios.2026.118933. 42314283

[79] M. Rezki and S. Tsujimura , “Hybrid Bioelectronic Interfaces With Supramolecularly Immobilized Redox Mediators for Ultra‐Stable and High‐Performance Enzyme Electrodes,” ACS Applied Materials & Interfaces 17, no. 31 (2025): 44101–44111, 10.1021/acsami.5c06219. 40690255

[80] M. Kalaj , K. C. Bentz , S. Ayala Jr. , et al., “MOF‐Polymer Hybrid Materials: From Simple Composites to Tailored Architectures,” Chemical Reviews 120, no. 16 (2020): 8267–8302, 10.1021/acs.chemrev.9b00575. 31895556

[81] S. H. Chen , Y. X. Li , P. H. Li , et al., “Electrochemical Spectral Methods for Trace Detection of Heavy Metals: A Review,” TrAC Trends in Analytical Chemistry 106 (2018): 139–150, 10.1016/j.trac.2018.07.005.

[82] N. Garg , A. Deep , and A. L. Sharma , “Recent Trends and Advances in Porous Metal‐Organic Framework Nanostructures for the Electrochemical and Optical Sensing of Heavy Metals in Water,” Critical Reviews in Analytical Chemistry 54 (2024): 1121–1145, 10.1080/10408347.2022.2106543. 35968634

[83] X. Chen , K. Geng , R. Liu , et al., “Covalent Organic Frameworks: Chemical Approaches to Designer Structures and Built‐in Functions,” Angewandte Chemie International Edition 59 (2020): 5050–5091, 10.1002/anie.v59.13. 31144373

[84] S. Seth and S. Jhulki , “Porous Flexible Frameworks: Origins of Flexibility and Applications,” Materials Horizons 8 (2021): 700–727, 10.1039/D0MH01710H. 34821313

[85] L. Jiao , Y. Wang , H. L. Jiang , and Q. Xu , “Metal–Organic Frameworks as Platforms for Catalytic Applications,” Advanced Materials 30 (2018): 1703663. 10.1002/adma.201703663 29178384

[86] B. Li , H. M. Wen , Y. Cui , W. Zhou , G. Qian , and B. Chen , “Emerging Multifunctional Metal–Organic Framework Materials,” Advanced Materials 28 (2016): 8819–8860, 10.1002/adma.v28.40. 27454668

[87] L. E. Kreno , K. Leong , O. K. Farha , M. Allendorf , R. P. Van Duyne , and J. T. Hupp , “Metal–Organic Framework Materials as Chemical Sensors,” Chemical Reviews 112 (2012): 1105–1125, 10.1021/cr200324t. 22070233

[88] R. Y. Hassan , “Advances in Electrochemical Nano‐Biosensors for Biomedical and Environmental Applications: From Current Work to Future Perspectives,” Sensors 22 (2022): 7539, 10.3390/s22197539. PMC9573286 36236638

[89] N. Sun , S. S. A. Shah , Z. Lin , Y. Z. Zheng , L. Jiao , and H. L. Jiang , “MOF‐Based Electrocatalysts: An Overview From the Perspective of Structural Design,” Chemical Reviews 125 (2025): 2703–2792, 10.1021/acs.chemrev.4c00664. 40070208

[90] C. Chen , L. Shen , B. Wang , et al., “Environmental Applications of Metal–Organic Framework‐Based Three‐Dimensional Macrostructures: A Review,” Chemical Society Reviews 54 (2025): 2208–2245, 10.1039/D4CS00435C. 39791318

[91] L. Sun , M. G. Campbell , and M. Dincă , “Electrically Conductive Porous Metal–Organic Frameworks,” Angewandte Chemie International Edition 55 (2016): 3566–3579, 10.1002/anie.v55.11. 26749063

[92] W. J. Li , M. Tu , R. Cao , and R. A. Fischer , “Metal–Organic Framework Thin Films: Electrochemical Fabrication Techniques and Corresponding Applications and Perspectives,” Journal of Materials Chemistry A 4 (2016): 12356–12369, 10.1039/C6TA02118B.

[93] X. Han , X. Lin , Y. Sun , L. Huang , F. Huo , and R. Xie , “Advancements in Flexible Electronics Fabrication: Film Formation, Patterning, and Interface Optimization for Cutting‐Edge Healthcare Monitoring Devices,” ACS Applied Materials & Interfaces 16 (2024): 54976–55010, 10.1021/acsami.4c11976. 39356954

[94] S. Joo and R. B. Brown , “Chemical Sensors With Integrated Electronics,” Chemical Reviews 108 (2008): 638–651, 10.1021/cr068113+. 18184017

[95] Z. Wang , J. Shin , J. H. Park , H. Lee , D. H. Kim , and H. Liu , “Engineering Materials for Electrochemical Sweat Sensing,” Advanced Functional Materials 31 (2021): 2008130.

[96] Y. Pan , J. Zhang , X. Guo , Y. Li , L. Li , and L. Pan , “Recent Advances in Conductive Polymers‐Based Electrochemical Sensors for Biomedical and Environmental Applications,” Polymers 16 (2024): 1597, 10.3390/polym16111597. PMC11174834 38891543

[97] J. L. Rowsell and O. M. Yaghi , “Metal–Organic Frameworks: A New Class of Porous Materials,” Microporous and Mesoporous Materials 73 (2004): 3–14, 10.1016/j.micromeso.2004.03.034.

[98] Y. Liu , K. He , G. Chen , W. R. Leow , and X. Chen , “Nature‐Inspired Structural Materials for Flexible Electronic Devices,” Chemical Reviews 117 (2017): 12893–12941, 10.1021/acs.chemrev.7b00291. 28991450

[99] L. Zhang and M. Fang , “Nanomaterials in Pollution Trace Detection and Environmental Improvement,” Nano Today 5 (2010): 128–142, 10.1016/j.nantod.2010.03.002.

[100] Z. Song , N. Cheng , A. Lushington , and X. Sun , “Recent Progress on MOF‐Derived Nanomaterials as Advanced Electrocatalysts in Fuel Cells,” Catalysts 6 (2016): 116, 10.3390/catal6080116.

[101] Z. Yin , Y. L. Zhou , M. H. Zeng , and M. Kurmoo , “The Concept of Mixed Organic Ligands in Metal–Organic Frameworks: Design, Tuning and Functions,” Dalton Transactions (Cambridge, England: 2003) 44 (2015): 5258–5275, 10.1039/C4DT04030A. 25687325

[102] P. Judeinstein and C. Sanchez , “Hybrid Organic–Inorganic Materials: A Land of Multidisciplinarity,” Journal of Materials Chemistry 6 (1996): 511–525, 10.1039/JM9960600511.

[103] W. Y. Gao , M. Chrzanowski , and S. Ma , “Metal–Metalloporphyrin Frameworks: A Resurging Class of Functional Materials,” Chemical Society Reviews 43 (2014): 5841–5866, 10.1039/C4CS00001C. 24676096

[104] R. Aliakbari , S. Ramakrishna , E. Kowsari , et al., “Scalable Preparation of MOFs and MOF‐Containing Hybrid Materials for use in Sustainable Refrigeration Systems for a Greener Environment: A Comprehensive Review as Well as Technical and Statistical Analysis of Patents,” Research on Chemical Intermediates 48 (2022): 2767–2819, 10.1007/s11164-022-04738-1.

[105] H. Y. Li , S. N. Zhao , S. Q. Zang , and J. Li , “Functional Metal–Organic Frameworks as Effective Sensors of Gases and Volatile Compounds,” Chemical Society Reviews 49 (2020): 6364–6401, 10.1039/C9CS00778D. 32749390

[106] S. P. Badwal , S. S. Giddey , C. Munnings , A. I. Bhatt , and A. F. Hollenkamp , “Emerging Electrochemical Energy Conversion and Storage Technologies,” Frontiers in Chemistry 2 (2014): 79, 10.3389/fchem.2014.00079. PMC4174133 25309898

[107] J. Wang , J. Wang , Z. Kong , K. Lv , C. Teng , and Y. Zhu , “Conducting‐Polymer‐Based Materials for Electrochemical Energy Conversion and Storage,” Advanced Materials 29 (2017): 1703044, 10.1002/adma.v29.45. 28922486

[108] E. Doustkhah , R. Hassandoost , A. Khataee , R. Luque , and M. H. N. Assadi , “Hard‐Templated Metal–Organic Frameworks for Advanced Applications,” Chemical Society Reviews 50 (2021): 2927–2953, 10.1039/C9CS00813F. 33481980

[109] K. Shen , X. Chen , J. Chen , and Y. Li , “Development of MOF‐Derived Carbon‐Based Nanomaterials for Efficient Catalysis,” ACS Catalysis 6 (2016): 5887–5903, 10.1021/acscatal.6b01222.

[110] Z. Yuan , H. Chai , Y. Huang , et al., “Porphyrin‐Engineered Metal− Organic Frameworks for Photo/Electrochemical Sensing: Preparation and Mechanisms,” Coordination Chemistry Reviews 527 (2025): 216385, 10.1016/j.ccr.2024.216385.

[111] J. Lee , D. Kim , H. Y. Ryoo , and B. S. Shin , “Sustainable Wearables: Wearable Technology for Enhancing the Quality of Human Life,” Sustainability 8 (2016): 466, 10.3390/su8050466.

[112] J. J. Fang , N. N. Yang , and E. Q. Gao , “Making Metal–Organic Frameworks Electron‐Deficient for Ultrasensitive Electrochemical Detection of Dopamine,” Electrochemistry Communications 89 (2018): 32–37, 10.1016/j.elecom.2018.02.014.

[113] B. Ma , H. Guo , M. Wang , et al., “Electrocatalysis of Cu‐MOF/Graphene Composite and Its Sensing Application for Electrochemical Simultaneous Determination of Dopamine and Paracetamol,” Electroanalysis 31 (2019): 1002–1008, 10.1002/elan.v31.6.

[114] S. A. Hira , S. Nagappan , D. Annas , Y. A. Kumar , and K. H. Park , “NO_2_‐Functionalized Metal–Organic Framework Incorporating Bimetallic Alloy Nanoparticles as a Sensor for Efficient Electrochemical Detection of Dopamine,” Electrochemistry Communications 125 (2021): 107012, 10.1016/j.elecom.2021.107012.

[115] J. Tang , Y. Liu , J. Hu , et al., “Co‐Based Metal‐Organic Framework Nanopinnas Composite Doped With Ag Nanoparticles: A Sensitive Electrochemical Sensing Platform for Simultaneous Determination of Dopamine and Acetaminophen,” Microchemical Journal 155 (2020): 104759, 10.1016/j.microc.2020.104759.

[116] S. Cheng , X. Gao , S. DelaCruz , et al., “In Situ Formation of Metal–Organic Framework Derived CuO Polyhedrons on Carbon Cloth for Highly Sensitive Non‐Enzymatic Glucose Sensing,” Journal of Materials Chemistry B 7 (2019): 4990–4996, 10.1039/C9TB01166H. 31411623

[117] A. Aziz , M. Asif , G. Ashraf , et al., “Advancements in Electrochemical Sensing of Hydrogen Peroxide, Glucose and Dopamine by Using 2D Nanoarchitectures of Layered Double Hydroxides or Metal Dichalcogenides. A review,” Microchimica Acta 186 (2019): 671, 10.1007/s00604-019-3776-z. 31489483

[118] D. Zhang , X. Zhang , Y. Bu , J. Zhang , and R. Zhang , “Copper Cobalt Sulfide Structures Derived From MOF Precursors With Enhanced Electrochemical Glucose Sensing Properties,” Nanomaterials 12 (2022): 1394, 10.3390/nano12091394. PMC9102815 35564103

[119] H. Li , N. Xiao , M. Jiang , J. Long , Z. Li , and Z. Zhu , “Advances of Transition Metal‐Based Electrochemical Non‐Enzymatic Glucose Sensors for Glucose Analysis: A Review,” Critical Reviews in Analytical Chemistry 55 (2025): 1099–1135, 10.1080/10408347.2024.2339955. 38635407

[120] L. Zhang , C. Ye , X. Li , et al., “A CuNi/C Nanosheet Array Based on a Metal–Organic Framework Derivate as a Supersensitive Non‐Enzymatic Glucose Sensor,” Nano‐Micro Letters 10 (2018): 28, 10.1007/s40820-017-0178-9. PMC6199074 30393677

[121] M. Zamani‐Kalajahi and S. Kurbanoglu , Metal Organic Framework Based Electrochemical Sensing Devices. in: Real‐Time Applications of Advanced Electrochemical Sensing Devices (IOP Publishing, 2024). 6‐1–6‐24.

[122] Z. Zhang , H. Ji , Y. Song , et al., “Fe(III)‐Based Metal–Organic Framework‐Derived Core–Shell Nanostructure: Sensitive Electrochemical Platform for High Trace Determination of Heavy Metal Ions,” Biosensors & Bioelectronics 94 (2017): 358–364, 10.1016/j.bios.2017.03.014. 28319903

[123] X. J. Huang and Y. K. Choi , “Chemical Sensors Based on Nanostructured Materials,” Sensors and Actuators B: Chemical 122 (2007): 659–671, 10.1016/j.snb.2006.06.022.

[124] G. Mukundan , M. Ravipati , and S. Badhulika , “Bimetallic Fe/Co‐MOF Dispersed in a PVA/Chitosan Multi‐Matrix Hydrogel as a Flexible Sensor for the Detection of Lactic Acid in Sweat Samples,” Microchimica Acta 191 (2024): 614, 10.1007/s00604-024-06687-5. 39305317

[125] H. Zhao , X. Tan , H. Chai , et al., “Recent Advances in Conductive MOF‐Based Electrochemical Sensors,” Chinese Chemical Letters 36, no. 8 (2025): 110571, 10.1016/j.cclet.2024.110571.

[126] L. Wang , H. Yang , L. Xu , C. Peng , and Y. Song , “A Novel Dopamine‐Imprinted Chitosan/CuCo_2_O_4_@carbon/Three‐Dimensional Macroporous Carbon Integrated Electrode,” Journal of Alloys and Compounds 817 (2020): 152771, 10.1016/j.jallcom.2019.152771.

[127] E. Asadian , S. Shahrokhian , and A. I. Zad , “Highly Sensitive Nonenzymatic Glucose Sensing Platform Based on MOF‐Derived NiCo LDH Nanosheets/Graphene Nanoribbons Composite,” Journal of Electroanalytical Chemistry 808 (2018): 114–123, 10.1016/j.jelechem.2017.10.060.

[128] J. Meng , M. Zahran , and X. Li , “Metal–Organic Framework‐Based Nanostructures for Electrochemical Sensing of Sweat Biomarkers,” Biosensors 14 (2024): 495, 10.3390/bios14100495. PMC11506703 39451708

[129] X. Li , Y. Zhang , L. Xu , et al., “Ultrasensitive Sensors Reveal the Spatiotemporal Landscape of Lactate Metabolism in Physiology and Disease,” Cell Metabolism 35 (2023): 200–211.e9, 10.1016/j.cmet.2022.10.002. PMC10560847 36309010

[130] Q. Wang , X. Wen , and J. Kong , “Recent Progress on Uric Acid Detection: A Review,” Critical Reviews in Analytical Chemistry 50 (2020): 359–375, 10.1080/10408347.2019.1637711. 31296022

[131] Y. Tang , Y. Liu , Y. Xia , F. Zhao , and B. Zeng , “Simultaneous Detection of Ovarian Cancer‐Concerned HE4 and CA125 Markers Based on Cu Single‐Atom‐Triggered CdS QDs and Eu MOF@isoluminol ECL,” Analytical Chemistry 95 (2023): 4795–4802. 10.1021/acs.analchem.3c00273 36867090

[132] S. Liu , Y. Chen , X. Cheng , G. Li , and Y. Hu , “Dual Enzyme‐Mimicking Bimetallic MOF for Selective SERS Detection of L‐DOPA in Human Serum Based on Cascade Catalytic Reaction,” Talanta 294 (2025): 128178, 10.1016/j.talanta.2025.128178. 40262351

[133] S. Yadav and P. Malhotra , “Metal–Organic Frameworks (MOFs) as Sensors for Environmental Monitoring,” in: Metal‐Organic Frameworks (MOFs) as Catalysts (Springer Nature, 2022), 393–409.

[134] S. Y. Kanti , I. Csóka , L. Adalbert , and O. Jójárt‐Laczkovich , “Analysis of the Renewed European Medical Device Regulations in the Frame of the Non‐EU Regulatory Landscape During the COVID Facilitated Change,” Journal of Pharmaceutical Sciences 111 (2022): 2674–2686, 10.1016/j.xphs.2022.07.011. 35872025

[135] M. V. Nikolic , V. Milovanovic , Z. Z. Vasiljevic , and Z. Stamenkovic , “Semiconductor Gas Sensors: Materials, Technology, Design, and Application,” Sensors 20 (2020): 6694, 10.3390/s20226694. PMC7700484 33238459

[136] K. He , Z. Li , L. Wang , et al., “A Water‐Stable Luminescent Metal–Organic Framework for Rapid and Visible Sensing of Organophosphorus Pesticides,” ACS Applied Materials & Interfaces 11 (2019): 26250–26260, 10.1021/acsami.9b06151. 31251555

[137] D. Aziz , S. Rafiq , P. Saini , et al., “Remote Sensing and Artificial Intelligence: Revolutionizing Pest Management in Agriculture,” Frontiers in Sustainable Food Systems 9 (2025): 1551460, 10.3389/fsufs.2025.1551460.

[138] R. Yousefi , S. Asgari , A. B. Dehkordi , et al., “MOF‐Based Composites as Photoluminescence Sensing Platforms for Pesticides: Applications and Mechanisms,” Environmental Research 226 (2023): 115664, 10.1016/j.envres.2023.115664. 36913998

[139] Y. Chen , W. Lu , M. Schröder , and S. Yang , “Analysis and Refinement of Host–Guest Interactions in Metal–Organic Frameworks,” Accounts of Chemical Research 56 (2023): 2569–2581, 10.1021/acs.accounts.3c00243. PMC10552526 37646412

[140] R. Kaur , D. Singh , A. Kumari , et al., “Pesticide Residues Degradation Strategies in Soil and Water: A Review,” International Journal of Environmental Science and Technology 20 (2023): 3537–3560, 10.1007/s13762-021-03696-2.

[141] S. Mao , J. Chang , H. Pu , et al., “Two‐Dimensional Nanomaterial‐Based Field‐Effect Transistors for Chemical and Biological Sensing,” Chemical Society Reviews 46 (2017): 6872–6904, 10.1039/C6CS00827E. 28933459

[142] A. S. Tsagkaris , J. Pulkrabova , and J. Hajslova , “Optical Screening Methods for Pesticide Residue Detection in Food Matrices: Advances and Emerging Analytical Trends,” Foods 10 (2021): 88, 10.3390/foods10010088. PMC7824741 33466242

[143] K. Vikrant , D. C. Tsang , N. Raza , B. S. Giri , D. Kukkar , and K. H. Kim , “Potential Utility of Metal–Organic Framework‐Based Platform for Sensing Pesticides,” ACS Applied Materials & Interfaces 10 (2018): 8797–8817, 10.1021/acsami.8b00664. 29465977

[144] S. Liu , J. Zhou , X. Yuan , et al., “A Dual‐Mode Sensing Platform Based on Metal–Organic Framework for Colorimetric and Ratiometric Fluorescent Detection of Organophosphorus Pesticide,” Food Chemistry 432 (2024): 137272, 10.1016/j.foodchem.2023.137272. 37657347

[145] D. D. Chronopoulos , H. Saini , I. Tantis , R. Zbořil , K. Jayaramulu , and M. Otyepka , “Carbon Nanotube Based Metal–Organic Framework Hybrids From Fundamentals Toward Applications,” Small 18 (2022): 2104628, 10.1002/smll.v18.4. 34894080

[146] P. Sabena and P. Shukla , “A Review on Recent Developments and Advances in Environmental Gas Sensors to Monitor Toxic Gas Pollutants,” Environmental Progress & Sustainable Energy 42 (2023): e14126, 10.1002/ep.v42.5.

[147] M. Ravichand , D. Ganesh , J. N. Madhuri , K. N. Bharath , D. S. Rani , and M. S. Kumar , “Analysis and Classification of Tweets for Sentiment Analysis on Twitter Dataset,” in 2025 3rd International Conference on Artificial Intelligence and Machine Learning Applications Theme: Healthcare and Internet of Things (AIMLA) , (IEEE, 2025), 1–8.

[148] H. Zhang and R. Srinivasan , “A Systematic Review of Air Quality Sensors, Guidelines, and Measurement Studies for Indoor Air Quality Management,” Sustainability 12 (2020): 9045, 10.3390/su12219045.

[149] R. A. Potyrailo , C. Surman , N. Nagraj , and A. Burns , “Materials and Transducers Toward Selective Wireless Gas Sensing,” Chemical Reviews 111 (2011): 7315–7354, 10.1021/cr2000477. PMC3212628 21899304

[150] D. A. Gkika , M. Chalaris , and G. Z. Kyzas , “Review of Methods for Obtaining Rare Earth Elements From Recycling and Their Impact on the Environment and Human Health,” Processes 12 (2024): 1235, 10.3390/pr12061235.

[151] Y. Fan , X. Zhu , H. Sui , H. Sun , and Z. Wang , “Design and Application of Toxic and Harmful Gas Monitoring System in Fire Fighting,” Sensors 19 (2019): 369, 10.3390/s19020369. PMC6358806 30658471

[152] R. Baron and J. Saffell , “Amperometric Gas Sensors as a Low Cost Emerging Technology Platform for Air Quality Monitoring Applications: A Review,” ACS Sensors 2 (2017): 1553–1566, 10.1021/acssensors.7b00620. 29025261

[153] A. Rehman and X. Zeng , “Methods and Approaches of Utilizing Ionic Liquids as Gas Sensing Materials,” RSC Advances 5 (2015): 58371–58392, 10.1039/C5RA06754E. PMC5683717 29142738

[154] D. Y. Nadargi , A. Umar , J. D. Nadargi , et al., “Gas Sensors and Factors Influencing Sensing Mechanism With a Special Focus on MOS Sensors,” Journal of Materials Science 58 (2023): 559–582, 10.1007/s10853-022-08072-0.

[155] J. Zhang , X. Liu , G. Neri , and N. Pinna , “Nanostructured Materials for Room‐Temperature Gas Sensors,” Advanced Materials 28 (2016): 795–831, 10.1002/adma.v28.5. 26662346

[156] W. F. Cheung , T. H. Lin , and Y. C. Lin , “A Real‐Time Construction Safety Monitoring System for Hazardous Gas Integrating Wireless Sensor Network and Building Information Modeling Technologies,” Sensors 18 (2018): 436, 10.3390/s18020436. PMC5856143 29393887

[157] H. Yuan , N. Li , W. Fan , H. Cai , and D. Zhao , “Metal–Organic Framework Based Gas Sensors,” Advancement of Science 9 (2022): 2104374, 10.1002/advs.v9.6. PMC8867161 34939370

[158] Z. Rahman and V. P. Singh , “The Relative Impact of Toxic Heavy Metals (THMs) (arsenic (As), Cadmium (Cd), Chromium (Cr) (VI), Mercury (Hg) and Lead (Pb)) on the Total Environment: An Overview,” Environmental Monitoring and Assessment 191 (2019): 419. 10.1007/s10661-019-7528-7 31177337

[159] G. Aragay , J. Pons , and A. Merkoçi , “Recent Trends in Macro‐, Micro‐, and Nanomaterial‐Based Tools and Strategies for Heavy‐Metal Detection,” Chemical Reviews 111 (2011): 3433–3458, 10.1021/cr100383r. 21395328

[160] D. N. Unal , S. Sadak , and B. Uslu , “A Review on Electrochemical and Optical Sensing Platform Based on Ionic Liquids for Different Molecules Determination,” Critical Reviews in Analytical Chemistry 53 (2023): 798–824, 10.1080/10408347.2021.1978055. 34632874

[161] N. Joshi , T. Hayasaka , Y. Liu , H. Liu , O. N. Oliveira Jr , and L. Lin , “A Review on Chemiresistive Room Temperature Gas Sensors Based on Metal Oxide Nanostructures, Graphene and 2D Transition Metal Dichalcogenides,” Microchim Acta 185 (2018): 213, 10.1007/s00604-018-2750-5. 29594538

[162] K. Jayaramulu , S. Mukherjee , D. M. Morales , et al., “Graphene‐Based Metal–Organic Framework Hybrids for Applications in Catalysis, Environmental, and Energy Technologies,” Chemical Reviews 122 (2022): 17241–17338, 10.1021/acs.chemrev.2c00270. PMC9801388 36318747

[163] U. Schubert , “Cluster‐Based Inorganic–Organic Hybrid Materials,” Chemical Society Reviews 40 (2011): 575–582, 10.1039/C0CS00009D. 20877873

[164] H. Khosropour , M. Keramat , F. Tasca , and W. Laiwattanapaisal , “A Comprehensive Review of the Application of Zr‐Based Metal–Organic Frameworks for Electrochemical Sensors and Biosensors,” Microchimica Acta 191 (2024): 449, 10.1007/s00604-024-06515-w. 38967877

[165] N. Agrawal , D. Bhagel , P. Mishra , D. Prasad , and E. Kohli , “Post‐Synthetic Modification of Graphene Quantum Dots Bestows Enhanced Biosensing and Antibiofilm Ability: Efficiency Facet,” RSC Advances 12 (2022): 12310–12320, 10.1039/D2RA00494A. PMC9027252 35480352

[166] Y. Cui , B. Li , H. He , W. Zhou , B. Chen , and G. Qian , “Metal–Organic Frameworks as Platforms for Functional Materials,” Accounts of Chemical Research 49 (2016): 483–493, 10.1021/acs.accounts.5b00530. 26878085

[167] A. Wang , Y. Ma , and D. Zhao , “Pore Engineering of Porous Materials: Effects and Applications,” ACS Nano 18 (2024): 22829–22854, 10.1021/acsnano.4c08708. 39152943

